# Structural Mapping and Functional Characterization of Zebrafish Class B G-Protein Coupled Receptor (GPCR) with Dual Ligand Selectivity towards GLP-1 and Glucagon

**DOI:** 10.1371/journal.pone.0167718

**Published:** 2016-12-08

**Authors:** Deena A. Oren, Yang Wei, Luce Skrabanek, Billy K. C. Chow, Thomas Mommsen, Svetlana Mojsov

**Affiliations:** 1 The Rockefeller University, New York, New York, United States of America; 2 Applied Bioinformatics Core, Weill Cornell Medical College, New York, New York, United States of America; 3 School of Biological Sciences, The University of Hong Kong, Hong Kong SAR, China; 4 Department of Biochemistry, University of Victoria, Victoria, British Columbia, Canada; Universidade de Vigo, SPAIN

## Abstract

GLP-1 and glucagon regulate glucose metabolism through a network of metabolic pathways initiated upon binding to their specific receptors that belong to class B G-protein coupled receptors (GPCRs). The therapeutic potential of glucagon is currently being evaluated, while GLP-1 is already used in the treatment of type 2 diabetes and obesity. Development of a second generation of GLP-1 based therapeutics depends on a molecular and structural understanding of the interactions between the GLP-1 receptor (GLP-1R) and its ligand GLP-1. There is considerable sequence conservation between GLP-1 and glucagon and between the hGLP-1R and human glucagon receptor (hGCGR), yet each receptor recognizes only its own specific ligand. Glucagon receptors in fish and frogs also exhibit ligand selectivity only towards glucagon and not GLP-1. Based on competitive binding experiments and assays of increase in intracellular cAMP, we demonstrate here that a GPCR in zebrafish (*Danio rerio*) exhibits dual ligand selectivity towards GLP-1 and glucagon, a characteristic not found in mammals. Further, many structural features found in hGLP-1R and hGCGR are also found in this zebrafish GPCR (zfGPCR). We show this by mapping of its sequence and structural features onto the hGLP-1R and hGCGR based on their partial and complementary crystal structures. Thus, we propose that zfGPCR represents a dual GLP-1R/GCGR. The main differences between the three receptors are in their stalk regions that connect their N-terminal extracellular domains (NECDs) with their transmembrane domains and the absence of loop 3 in the NECD in zfGLP-1R/GCGR. These observations suggest that the interactions between GLP-1 and glucagon with loop 3 and the stalk regions may induce different conformational changes in hGLP-1R and hGCGR upon ligand binding and activation that lead to selective recognition of their native ligands.

## Introduction

Selective ligand recognition by G-protein coupled receptors (GPCRs) is critical for the initiation of different intracellular pathways that give rise to specific physiological responses. In mammals the specificity of ligand-receptor interactions ensures an accurate control of the hormonal regulation of metabolic processes. One example illustrating these principles is the regulation of metabolic pathways by the glucagon-secretin family of peptide hormones and neurotransmitters. They are grouped together on the basis of their sequence similarities and ability to stimulate intracellular cAMP after binding to their G-protein coupled receptors (GPCR). Within this family of peptides, the highest sequence similarities are found between glucagon and glucagon-like peptides 1 and 2 (GLP-1 and GLP-2) which are encoded by the glucagon gene [1] and released in a tissue-specific manner by proteolytic processing from their precursor protein preproglucagon [2], [3]. In mammals, glucagon and GLP-1 exert opposite effects on circulating glucose levels. Glucagon increases glucose levels by stimulating glycogenolysis [4, 5] and gluconeogenesis [6] from the liver, while GLP-1 decreases circulating glucose levels indirectly by stimulating insulin release from the pancreas [7] [8] [9]. Increased insulin levels lead to the uptake of glucose from the circulation by the muscle, liver and adipose tissues. These physiological processes are initiated after binding of glucagon to its specific receptor expressed in the liver [10] and GLP-1 binding to its specific receptor expressed in the pancreas [11] [12], with each receptor having high affinity only towards its own ligand [13] [14].

**Fig 1:**
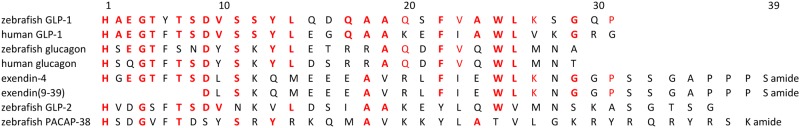
Alignment of the amino acid sequence of zfGLP-1 with sequences of hGLP-1, zebrafish glucagon, human glucagon, exendin-4, exendin(9–39), zfGLP-2 and zebrafish PACAP-38. Identical amino acids are shown in red. Numbering of hGLP-1 starts at 1 with the amino terminal histidine, corresponding to the biologically active hGLP-1(7–37) and hGLP-1(7–36)amide, to be consistent with histidine 1 in zfGLP-1 (see Materials and Methods).

Receptors for GLP-1 (GLP-1R) and glucagon (GCGR) belong to the class B GPCRs that mediate the physiological effects of the peptides within the glucagon-secretin peptide family [15, 16]. Their structures are characterized by large amino terminal extracellular domains which are about 140 amino acids long and an intracellular core consisting of a cytoplasmic domain and seven transmembrane helices (7TMs) connected through three extracellular and three intracellular loops. Functional characterization of the class B GPCRs demonstrated that some, including the vertebrate GCGRs, exhibit ligand binding specificity only towards their native ligand glucagon [13, 17], [18, 19] while others, can bind several physiologically relevant peptide ligands (e.g., corticotropin releasing factor receptors (CRF- R) [20], VPAC receptors for pituitary adenylyl cyclase activating polypepide (PACAP) and vasoactive intestinal polypeptide (VIP)[21] [22]). In addition to its own endogenous GLP-1 ligand, GLP-1R, binds the peptide exendin-4 [23] [24], a peptide exclusively found in the saliva of a lizard [25]. GLP-1 and exendin-4 are homologous peptides and show about 50% sequence identity (Fig 1), but bind to the GLP-1R with similar binding affinities and stimulate intracellular cAMP to a similar degree [23].

A “two domain” hypothesis has been proposed to explain the mechanism of binding of peptide ligands to their class B GPCRs [26], [27]. In this model, the carboxyl terminal end of the ligand binds to the N-terminal extracellular domain of the receptor (NECD). This interaction allows the N-terminal end of the ligand to position itself within the membrane spanning intracellular core of the receptor inducing a conformational change of the receptor and thereby receptor activation. It has also been proposed that the α-helical conformation of the ligands is important for their recognition by the NECD [27].

There is a growing interest in understanding the mechanism of interaction of the class B GPCRs with their ligands, especially for peptides with therapeutic potential [28] such as GLP-1 and exendin-4, both used clinically for the treatment of individuals with type 2 diabetes. GLP-1 was also recently approved as a treatment for obesity. Crystal structures of NECDs have been solved for several class B GPCRs [29] [30–34] including GLP-1R [35, 36]. They have contributed significantly to our understanding of the initial steps involved in the ligand-receptor interactions. The first detailed information about the nature of the interactions of the TMs in class B GPCRs became available from the crystal structures of the intracellular seven TMs (7TMs) of the hGCGR [30] (PDB entry 4L6R) and human corticotropin receptor type 1 (hCRF-R1) [31] (PDB entry 4K5Y). Some of the interactions are conserved between hGCGR and hCRF-R1 suggesting that they may represent common structural features of the transmembrane domains of class B GPCRs [37].

Our approach to understanding the selectivity of ligand recognition by the receptors for the gluco-regulatory peptides, GLP-1 and glucagon, has been to functionally characterize the ligand selectivity of GCGRs and GLP-1Rs from non-mammalian vertebrates and to compare them to the ligand selectivity of their mammalian counterparts. The rationale behind this approach was based on the observation that the effect of GLP-1 on glucose metabolism in teleost fish was similar to that of glucagon [38–40] i.e., GLP-1 (from both human and fish) stimulated gluconeogenesis of fish hepatocytes. In the absence of genomic sequences from teleost fish at that time, these observations raised the question whether the gluco-regulatory actions of GLP-1 and glucagon in fish involved a single receptor or two separate specific receptors, as in mammals, each recognizing its own endogenous ligand.

We demonstrated that the glucagon receptors from the goldfish *Carrassius auratus* (gfGCGR) [18] and the frog *Rana tigrina regulosa* (now *Holobatrachus tigrinus*) (fGCGR) [19] have ligand specificities only towards glucagon and not GLP-1, analogous to their mammalian counterparts. Our initial characterization of a GPCR isolated from zebrafish (zfGPCR) demonstrated similar ligand specificity towards zebrafish and human GLP-1’s which differ in 10 out of 31 amino acids (Fig 1), as determined by similar IC_50_ values obtained in competitive binding experiments [41]. This recombinant zfGPCR also bound exendin-4, an agonist of rat GLP-1R (rGLP-1R) and hGLP-1R, with an IC_50_ in the low nM range, similar to the IC_50_ values obtained for rGLP-1R [23] and hGLP-1R [41]). Based on these results, we concluded that we had isolated and characterized a zebrafish homolog of the hGLP-1R [41].

Here we show by extensive sequence and structural mapping of this zfGPCR onto the hGLP-1R and hGCGR based on their partial and complementary crystal structures [35, 36] (PDB entry 3C59 and 3IOL, respectively), [32] (PDB entry 4ERS) and [30] (PDB entry 4L6R) that a great number of structural features found in hGLP-1R and hGCGR are also found in this zfGPCR. Therefore, we need to modify our initial assignment of the zfGLP-1R [41] to be instead a dual zfGLP-1R/GCGR. The dual zfGLP-1R/GCGR displays, in competitive binding experiments and assays of increase of intracellular cAMP, ligand selectivity towards both GLP-1 and glucagon not found in mammalian GLP-1Rs and GCGRs. There are also major differences between the three receptors, namely differences in the stalk region that connects the NECD to the TM domain, and the absence of loop 3 in the NECD of the zfGPCR. We propose that loop 3 and the stalk regions together may contribute to the specific recognition of hGLP-1R and hGCGR of their native ligands.

## Materials and Methods

### Synthetic peptides

Sequences of the synthetic peptides used in the functional experiments are shown in Fig 1. Synthetic hGLP-1(7–36)amide (referred throughout as hGLP-1), synthetic human glucagon, exendin-4 and exendin(9–39) were purchased from Bachem (Torrence, CA). zfGLP-1, zebrafish glucagon and zfGLP-2 were synthesized by the Rockefeller University Proteomics Facility. Zebrafish PACAP-38amide was synthesized by the Protein and Carbohydrate structure facility at the University of Michigan. The homogeneity of all peptides used in the functional experiments was checked by HPLC and mass spectroscopy and was >99%.

### Numbering of amino acids in hGLP-1 and designation of individual amino acids in the sequences of hGLP-1, exendin-4, exendin(9–39) and glucagon

We here use GLP-1 to refer to both GLP-1(7–37) and GLP-1(7–36)amide. The N-terminal histidine in GLP-1(7–37) is labeled as 1 instead of 7 to be consistent with the alignment with the sequence of zfGLP-1. The biologically active hGLP-1 is 31 residues long (GLP-1(7–37)) or 30 residues long when amidated at its carboxyl terminal (GLP-1(7–36)amide).

Individual amino acids in hGLP-1 are denoted with *, in exendin-4 and exendin(9–39) with ** and in human glucagon with ***.

### Numbering of amino acids in the multiple sequence and structural alignments between zfGPCR (dual zfGLP-1R /GCGR) and hGLP-1R and hGCGR

We refer in the Introduction, the Materials and Methods and in the Results to the class B GPCR in zebrafish as zfGPCR to indicate the incomplete characterization of receptor’s ligands. After our functional experiments showed that this zfGPCR has ligand selectivity towards both GLP-1 and glucagon we refer to it as dual zfGLP-1R/GCGR to highlight its selectivity towards both GLP-1 and glucagon.

Sequence and structural mapping of the zfGPCR with hGLP-1R and hGCGR was based on the crystal structures of the NECD of hGLP-1R in complex with hGLP-1 (PDB entry 3IOL) [36] and in complex with exendin(9–39) (PDB entry 3C59), [35], the NECD of hGCGR in complex with Fab fragments of several monoclonal antibodies that block glucagon binding and inhibit basal receptor activity (PDB entry 4ERS) [32] and the 7TM crystal structure of hGCGR (PDB entry 4L6R), [30]. To compare the structural features of the three receptors, the NECDs including the stalk regions (see Results section) were numbered according to the crystal structures of NECD of hGLP-1R in complex with hGLP-1 [36] (PDB entry 3IOL) or in complex with exendin(9–39) [35] (PDB entry 3C59). Numbering in the 7TM domains (see Results section) is according to the 7TM crystal structure of hGCGR [30] (PDB entry 4L6R). Individual residues in the 7TM domains were numbered according to the numbering system used by Wootten et al. [42]. It is a modification of the Ballesteros-Weinstein numbering used in the family A GPCRs [43], where the first number in the superscript denotes the helix (1–7) and the second the residue position relative to the most conserved position, which is assigned the number 50.

### Graphic presentations

The snake diagram of the zfGPCR (zfGLP-1R/GCGR) was based on the output of the residue-based diagram editor RbDe [44]. Residues are colored using the GPCRDB coloring scheme, where residues with similar physicochemical properties are colored with the same color.

All 3D structures were visualized using the PyMOL Molecular Graphics System, Version 1.7.7.4 Schrödinger, LLC [45].

### Calculation of secondary structures

A helical wheel diagram [46] was used to demonstrate the amphiphilic nature of the helix in the region of amino acid 32–52 in the sequences of hGLP-1R, zfGPCR and hGCGR.

### Competitive binding experiments

Competitive binding experiments were performed with the recombinant zfGPCR expressed in COS-7 cells, as described previously [12, 18, 19]. Transient transfections of pcDNA3 vector containing recombinant zfGPCR into COS-7 cells were performed as described below for the stimulation of intracellular cAMP. Cells were grown in 100-mm plates to confluence and 24 hr after transfection they were trypsinized and transferred to 24 well plates (Biocore, Becton Dickinson). 24–48 hr later each peptide (pM to μM) was added in triplicate wells followed by the addition of an aliquot (100,000 cpm/well) of either ^125^I-hGLP-1(7–36)amide or ^125^I-exendin(9–39) (each radioactive peptide tracer at 2200 mCi/mmol receptor grade, NEN Life Science Products, Boston, MA). Peptides, radioiodinated ^125^I-tracer and cells were incubated for 16–18 h at 4°C. After incubation cells were washed twice with ice-cold PBS, lysed with 1N NaOH and radioactivity measured in a γ-counter. A single dose-displacement curve for each peptide was obtained in a single 24-well plate.

Each dose-dependent displacement curve shown in the competitive binding experiments with ^125^I-GLP-1(7–36)amide as a tracer represents an average of three rounds of transfections for hGLP-1 (n = 3), two for exendin-4, exendin(9–39) and zebrafish glucagon (n = 2). Dose-response curves for zfGLP-1, zfGLP-2 and zebrafish PACAP-38 were obtained from one transfection. Non-specific-binding was determined in the presence of 1μM of each peptide used to characterize the dose-dependent displacement curves for the recombinant zfGPCR, except for zfGLP-2 and zebrafish PACAP-38 where zfGLP-1 at 1μM was used for the non-specific binding as a positive control because in preliminary experiments we observed that zfGLP-2 and zfPACAP-38 did not displace the binding of 125I -GLP-1(7–36)amide at 1μM concentration. Lower rounds of transfections were performed in experiments that replicated our published results from the competitive binding experiments with the displacement of ^125^I-hGLP-1(7–36)amide binding to zfGPCR [41] and thus represented positive controls for these studies.

In the competitive binding experiments when ^125^I-exendin(9–39) was used as a tracer, results represent an average of n = 4 separate rounds of transfections for zfGLP-1, n = 3 for hGLP-1, zebrafish glucagon, human glucagon and exendin-4 and n = 5 for exendin(9–39). Non-specific binding was determined in the presence of 1μM of each peptide.

IC_50_ values represent an average of n experiments as described above and were calculated by the Prism 4 software [47]. Displacement curves from the competitive binding experiments were plotted by Origin 9 software (OriginLab). Error bars are shown for data points in the displacement curves obtained in two or more rounds of transfections.

### Measurement of intracellular cAMP levels

The ability of different peptides to stimulate the increase in intracellular cAMP levels following their binding to the recombinant zfGPCR transiently expressed in COS-7 cells was measured using methods that we developed for non-mammalian G-protein coupled receptors [18]. In brief, COS-7 cells were grown to confluence in 100-mm plates at 37°C and transfected with the pcDNA3 vector containing the recombinant zfGPCR using the TransFast transfection reagent (Promega, Madison, WI) at a 1:1 ratio of plasmid DNA (25 ug) to transfection reagent (75 μL). After 24 h cells were trypsynized and transferred to 24-well plates (Biocore, Becton-Dickinson, Franklin Lakes, NJ). Peptides (pM-μM) were added 24–48 h later. Cells were equilibrated prior to the addition of peptides with the assay buffer (DMEM, 0.5% BSA, 20mM HEPES, 1mM 1-methyl-3-isobutylxanthine, 0.1mM phenylmethylsulfonyl fluoride, pH 7.4) for 20 min at 37°C. Each peptide concentration was added in triplicate wells for 20 min at 37°C. cAMP dose-response curves for each tested peptide were obtained in a single 24-well plate. In all experiments, forskolin (100 nM) (Sigma-Aldrich, St.Louis, MO) was added as a positive control, also in triplicate wells, in the same 24-well plate as the tested peptide. After incubation, the media was removed and cells were lysed by the addition of cold ethanol (1 mL per well). Cell debris was pelleted by centrifugation (10,000 g) for 10-min, and supernatants were dried using a vacuum concentrator. Samples were resuspended in an assay buffer provided by the manufacturer and cAMP levels were quantified by the enzyme immunoassay kit (Cayman Chemicals, Ann Arbor, MI) according to the manufacturer’s instructions. The basal concentration of cAMP (in the absence of peptides) was taken as 1.00, and was in the range of 9–16 pmol/well. Results are presented as a fold-increase over basal.

Each dose-response curve with the recombinant zfGPCR transiently transfected into COS-7 cells represents an average of n = 9 separate rounds transfections for zfGLP-1, n = 5 for hGLP-1, zebrafish glucagon, and human glucagon, n = 3 for exendin-4, n = 4 for exendin(9–39), n = 6 for zfGLP-2, and n = 2 for zebrafish PACAP-38. Results were plotted by the Origin 9 software (OriginLab), and EC_50_ values were calculated by Prism 4 software [47] as described below.

### Presentation of the data and statistical analysis

Results from the functional experiments with recombinant zfGPCR transiently expressed in COS-7 cells were analyzed by the Prism 4 software using a four-parameter logistic sigmoidal curve fit model [48]. Data points represent mean +/- SEM.

The IC_50_ values, defined to represent concentration of peptides that inhibit the specific binding by 50%, were calculated from the competitive binding curves. They are shown together with the 95% confidence intervals (CIs). The F-test was used to compare the IC_50_ value for zfGLP-1 with the IC_50_ values for the other tested peptides obtained in the competitive binding experiments shown when ^125^I-hGLP-1(7–36)amide or ^125^I-exendin (9–39) were used as tracers. Following conditions were used for the curve fitting: (i) in the normalization step y = 0%was set to be 0, y = 100% was set to be 100; (ii) constrained curve-fit parameters were set to be equal to 0.00 on the bottom, 100 on top; (iii) IC_50_ was not constrained; (iv) the selected option in the non-linear regression curve fit was intended on finding out whether the best-fit of a selected parameter (IC_50_) differs between two sets.

The EC_50_ values, representing peptide concentrations that give 50% of the maximum intracellular cAMP response were calculated by the Prism 4 software [47] from the midpoints on the cAMP dose-response curves. They are shown together with the 95% confidence intervals (CIs).

Comparison of EC_50_ values calculated from the zfGLP-1 cAMP dose-response curve with the EC_50_ calculated from the cAMP dose-response curves for each of the tested peptides were performed by the F-test from the Prism 4 software which compares the fitted mid-point (EC_50_) from the data sets of the two dose-response curves under comparison. Conditions of analysis were as follows: (i) in the normalization step y = 0% was set to be 1 (the basal cAMP concentration taken as 1); y = 100% was set to be the largest value in each data set; (ii) constrained curve-fit parameters were set to be equal to 0.00 on the bottom, 100 on top; (iii) EC_50_ was not constrained; (iv) the selected option in the non-linear regression curve fit was intended on finding out whether the best-fit of a selected parameter (EC_50_) differs between two sets.

Difference between the curves (corresponding to IC_50_ and EC_50_, respectively) are represented by the P-values, where P <0.05 is statistically significant.

## Results

**Fig 2:**
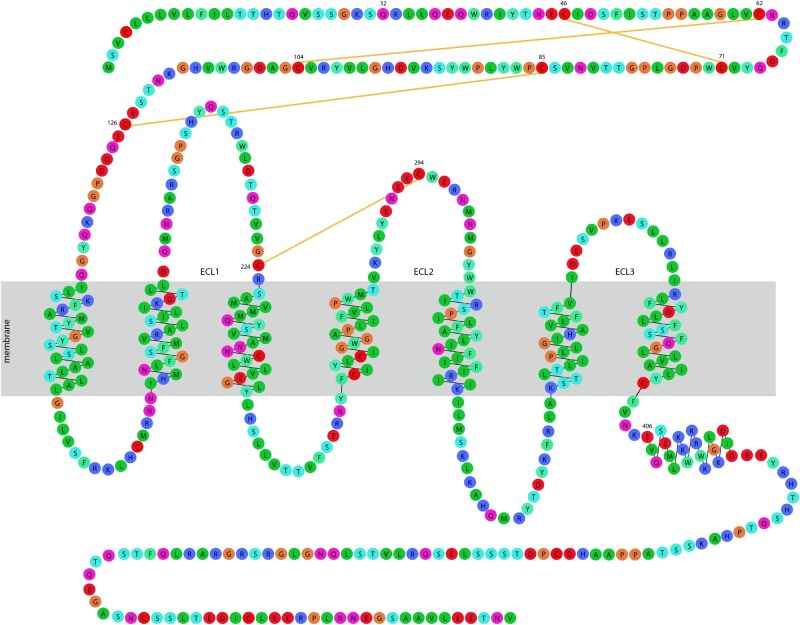
Snake diagram of the zfGPCR (dual zfGLP-1R/GCGR). Amino acids are numbered in the N-terminal extracellular domain (NECD) according to the numbering in the crystal structure of the NECD of hGLP-1R in complex with hGLP-1 (PDB entry 3IOL) [36]. Numbering of amino acids in the 7TM domain and the C-terminal cytoplasmic domain is according to the numbering in the 7TM crystal structure of hGCGR (PDB entry 4L6R) [30]. The following amino acids are numbered: (i) position 32 (glutamine) at the beginning of the predicted amphiphilic helix in zfGPCR corresponding to Leu32 at the beginning of the amphiphilic helix in hGLP-1R and Met32 at the beginning of the amphiphilic helix of hGCGR (see Fig 3 and S1 Fig); (ii) the eight cysteine residues forming the four disulfide bonds as indicated by yellow lines: (iii) glutamic acid in the cytoplasmic domain at position 406 at the beginning of helix 8 identified in the 7TM crystal structure of hGCGR (PDB entry 4L6R) [30]. Residues with similar physicochemical properties are colored with identical colors according to the residue-based diagram editor RbDe for GPCRs [44].

We had initially characterized the zfGPCR as a homolog of the hGLP-1R (Fig 2) as it exhibited similar binding specificities towards zebrafish and human GLP-1 peptides, as determined by their IC_50_s of 2 nM and 0.9 nM, respectively [41]. Furthermore, exendin-4, an agonist of hGLP-1R, displaced in competitive binding experiments the binding of ^125^I-hGLP-1(7–36) amide to the zebrafish receptor with an IC_50_ of 0.9 nM, which is within a similar concentration range as the one determined in competitive binding experiments with recombinant hGLP-1R [41] and rGLP-1R [23]. However, subsequent phylogenetic analysis showed that it belongs to the vertebrate glucagon receptor family, a distinct group from the mammalian GLP-1Rs [18] [49–51]

To further characterize the zfGPCR we compared its sequence with the sequence of hGLP-1R and hGCGR and mapped its structural features to those obtained from the crystal structures of its homologs in the Protein Data Bank.

### Mapping of the structural features of the NECD of hGLP-1R to the sequence of the NECD of zfGPCR

**Fig 3:**
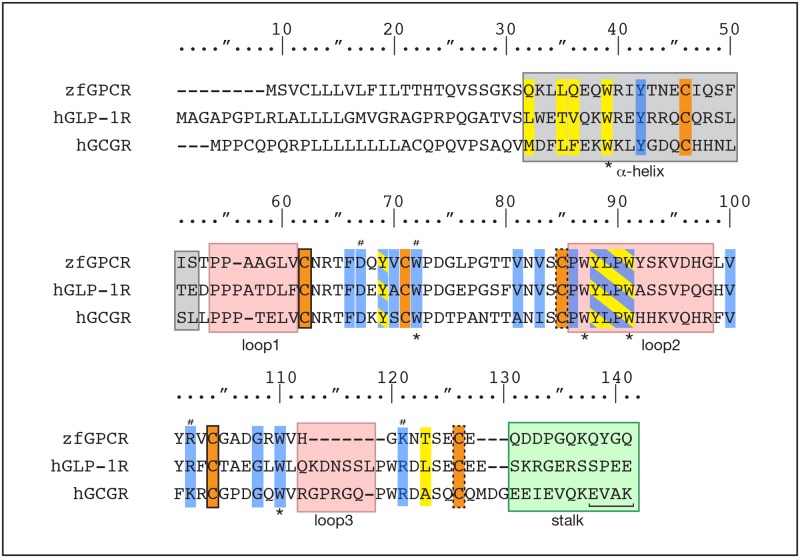
Multiple sequence alignment of the N-terminal extracellular domains (NECD) of zfGPCR, hGLP-1R and hGCGR. Numbering of amino acids is according to the numbering in the crystal structure of the NECD of hGLP-1R in complex with hGLP-1 (PDB entry 3IOL) [36]. The α-helical region is shaded grey, loops are colored pink, and the stalk region is shaded green. Note the absence of loop 3 in zfGPCR. The interhelical salt bridge in the α-helical stalk region in hGCGR identified in the 7TM crystal structure of hGCGR (PDB entry 4L6R) [30] is indicated at the end of the α- helical stalk region by a line. Residues with stabilizing functions are colored blue. Residues forming the exendin(9–39) hydrophobic binding pocket in the hGLP-1R NECD [35] (PDB entry 3C59) are colored in yellow. Residues which are both part of the binding pocket and have stabilizing functions are colored with blue and yellow hatching. Residues that are part of the hydrogen bond network in hGLP-1R coordinated by Asp67 [36] (PDB entry 3IOL) [35] (PDB entry 3C59) are marked with a hash sign. Cysteines are colored orange and paired cysteines are denoted by similar outlines (Fig 2). Conserved tryptophan residues are highlighted with an asterisk below the alignment.

Sequence and structural analyses of the NECD of zfGPCR with the corresponding regions in hGLP-1R (Fig 3) suggest that it contains the common structural fold of hGLP-1R, also found in all class B GPCRs [29, 35, 36]. Thus, the six cysteine residues forming the three disulfide bonds (Cys46-Cys71, Cys62-Cys104, Cys85-Cys126, orange Fig 3) are found in the corresponding positions in the sequence of the zfGPCR. [The numbering of amino acids in the NECD of these receptors follows the numbering in the crystal structures of the NECD of hGLP-1R either in complex with hGLP-1 [36] (PDB entry 3IOL) or with exendin(9–39) [35] (PDB entry 3C59).] Also conserved (blue, Fig 3) are the residues that play a central role in stabilizing the core of class B GPCR structures (Asp67, Trp72, Pro86, Arg102, Gly108, Trp110, and Arg121) [35, 36]. Additional conserved amino acids (blue Fig 3) include Tyr42, Phe66, Tyr69, Val81, Val83, Tyr88, Leu89, Pro90, Trp91 and Val100 which we and others postulate to play a role in the intramolecular interactions of the NECD that are specific for the GPCRs for the glucagon-secretin peptide family [35]. In zfGPCR Ala 70 is substituted with valine and Arg 121 with lysine (Fig 3), both conservative substitutions. Five of the seven Trp residues (Trp39, Trp72, Trp87, Trp91, Trp110, starred, Fig 3) found in the human and rat NECDs of GLP-1Rs are conserved in the sequence of the zfGPCR. [52, 53]

Crystal structures of the NECD of hGLP-1R in complex with hGLP-1 [36] or exendin(9–39) antagonist [35] also show that residues 32 to 52 form an α-helix (grey shading, Fig 3) terminated by three successive prolines in positions 54–56. Leu32 at the beginning of the helix is important for binding to exendin-4, but not to hGLP-1 [36] [54] and is substituted with glutamine in the zfGPCR. At the end of the helix there are two proline residues in the zfGPCR, Pro54-Pro55 (Fig 3).

**Fig 4:**
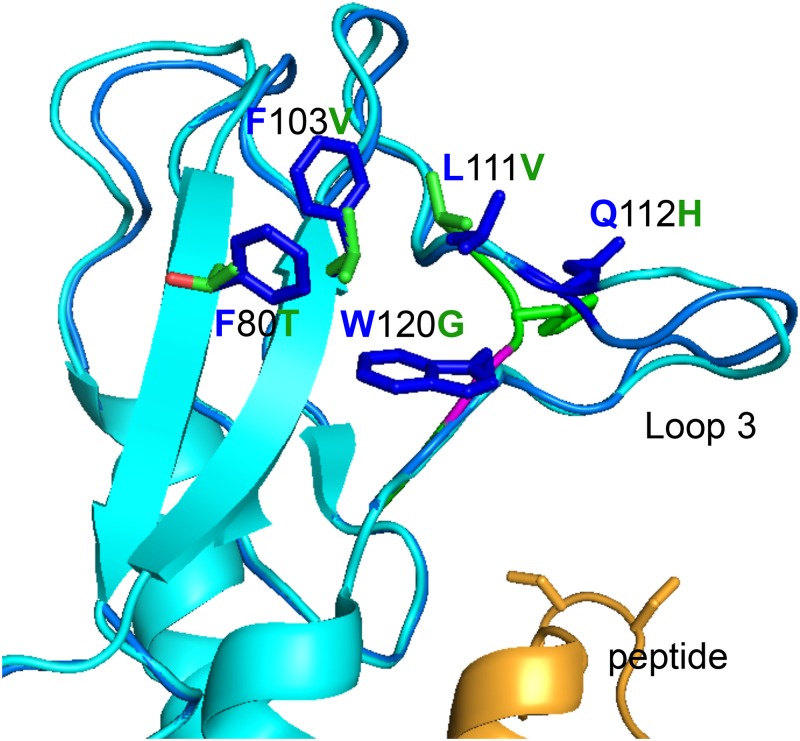
Amino acids in the hGLP-1R NECD hydrophobic cluster are primarily aromatic (blue color) while the corresponding residues in zfGPCR (green and magenta colors) are aliphatic (Fig 3), but likely do not affect the direct interaction of zfGPCR with exendin(9–39) (gold) or hGLP-1 (not shown) peptides. The conserved Tyr101 is not shown on the diagram for emphasis on differences. Cyan color represents the structure of the hGLP-1R NECD as bound to exendin(9–39) [35] (and PDB entry 3C59), or in light blue as bound to hGLP-1 [36] (and PDB 3IOL), and green the predicted zfGPCR structure based on the structure of the hGLP-1R NECD in complex with exendin(9–39) [35] (PDB entry 3C59). Backbone of residue Gly120 in zfGPCR (instead of Trp120 in hGLP-1R) is shown in magenta to highlight the lack of side chain. Note the absence of loop 3 in zfGPCR. Gln112 in hGLP-1R is a histidine in zfGPCR (Fig 3) maintaining similar hydrophobicity characteristics.

Loop 3 in hGLP-1R is absent in the sequence of the zfGPCR, while Trp120 in hGLP-1R is substituted with glycine in zfGPCR (Fig 3). In the crystal structures of the NECD of hGLP-1R in complex with hGLP-1 [36] and in complex with exendin(9–39) [35]Trp120 does not make contacts with either the GLP-1 or exendin(9–39), respectively, and therefore substitution of Trp120 with glycine and absence of loop 3 in zfGPCR should not affect its interactions with GLP-1 or exendin(9–39) (Fig 4). Trp120 plays a structural role in the NECD of hGLP-1R by participating in a hydrophobic cluster with Phe80, Tyr101, Phe103 and Leu111. In the zfGPCR sequence, Phe80 is substituted with threonine, Tyr101 is conserved, Phe103 is substituted with valine and Leu111 is substituted with valine (Fig 3). The amino acids in zfGPCR form an aliphatic hydrophobic cluster as compared to the aromatic nature in hGLP-1R (Fig 4).

**Fig 5:**
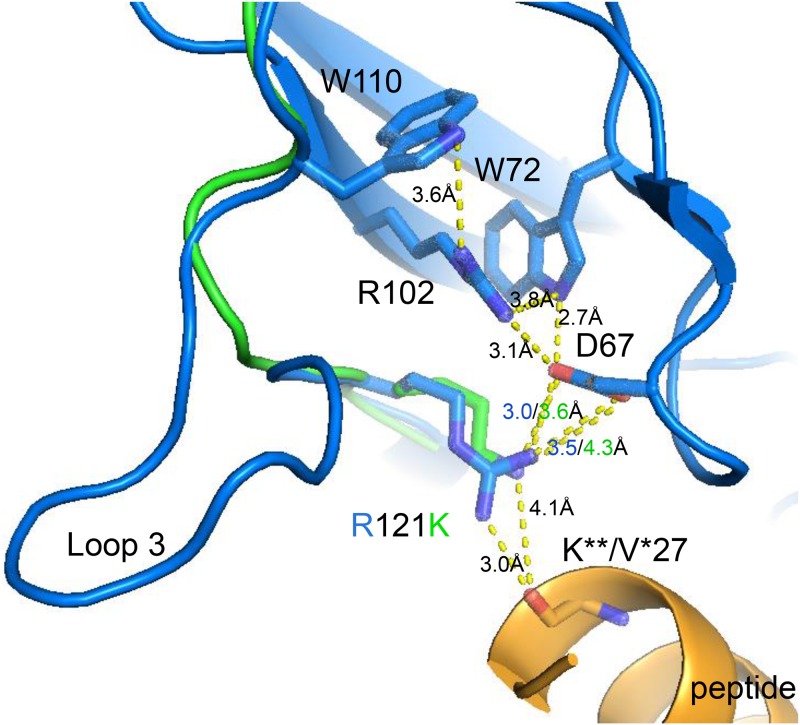
Substitution of Arg121 with lysine in zfGPCR and the absence of loop 3 should not affect the hydrogen bonding and the salt bridge network found in the crystal structures of the NECD of hGLP-1R in complex with exendin(9–39) or GLP-1 The hydrogen bond interaction between Arg121 in hGLP-1R with the main chain oxygen of position 27 in hGLP-1 (3.0Å) and exendin(9–39) (2.8Å, not shown) can be maintained by the conserved substitution of arginine with lysine in zfGPCR (Fig 3). Light blue ribbon diagram represents the structure of the hGLP-1R NECD bound to GLP-1 [36] (PDB entry 3IOL) and green the predicted structure of zfGPCR. Lys121 in zfGPCR was modeled from the structure of the hGLP-1R NECD in complex with hGLP-1 [36] (PDB entry 3IOL) by removing the missing residues and closing the gap with PyMOL’s sculpting module.

The crystal structures of the hGLP-1R NECD also show that Arg121 is important for binding but not specificity of interactions with exendin(9–39) and hGLP-1. The side chain of Arg121 forms a hydrogen bond with the backbone carbonyl of Lys**27 in exendin(9–39) and the backbone of Val*27 in hGLP-1 (Fig 1). In the crystal structure of the NECD of hGLP-1R in complex with hGLP-1 [36] in addition to the above interaction with Arg121 through its backbone, Val*27 in hGLP-1 also makes hydrophobic contacts with Tyr69 (conserved in zfGPCR) and Leu123. This specific interaction between hGLP-1 and the hGLP-1R NECD enables the direct salt bridge formation between Asp67 and Arg102 and preserves the integrity of the hydrogen bond network. In the zfGPCR, Arg121 is substituted with a lysine and Leu123 with a threonine, both conservative substitutions (Fig 3). Therefore, as shown on Fig 5, similar interactions between Lys121 in zfGPCR and the backbone of exendin(9–39) and hGLP-1 (Fig 1) could be maintained.

### Most amino acids in exendin(9–39) involved in the interactions with the hGLP-1R NECD are conserved in the NECD of zfGPCR

The structural and sequence comparisons between zfGPCR and hGLP-1R described earlier show almost complete conservation of amino acids that have been identified to be important for the interactions of exendin(9–39) with the NECD of hGLP-1R (Fig 3). In addition to the conservation of the hydrogen bond network where Asp67 interacts indirectly via a water molecule with the side chain of Arg102 and directly with the side chains of Trp72 and Arg121 the following amino acids and sequences are conserved: (i) Trp39 and Trp91 which are positioned directly in the hydrophobic interface with exendin(9–39) and are protected from solvent by exendin(9–39) binding; (ii) Pro90 which, with Trp91, is directed at the peptide’s hydrophobic face involving Val**19 and Ile**23 in exendin(9–39) (Fig 1). (iii) Glu127 which interacts through its side chain with the side chain of Lys**27 in exendin(9–39) (Fig 1). (iv) Tyr88 of loop 2 (pink color in Fig 3) which is positioned between Leu**26 of exendin(9–39) and Pro86 [35].

Some of the amino acids in hGLP-1R engaged in hydrophilic interactions with amino acids in exendin(9–39) are substituted in the zfGPCR. These substitutions could moderately modulate the binding and/or specificity of the interactions. They are: (i) Glu128, substituted with glutamine, whose side chain forms a salt bridge to the side chain of Arg**20 in exendin(9–39) (Fig 1), and (ii) Glu68, substituted with glutamine, which forms a hydrogen bond with the side chain of Ser**32 in exendin(9–39) (Fig 1). Residue 32 in the peptide only exists in exendin-4 and exendin(9–39). Arg121 is a lysine in zfGPCR (Figs 3 and 5), a substitution that maintains the hydrogen binding to the main chain oxygen of the Val**27 in exendin(9–39) (Figs 1 and 5).

### Mapping of the structural features of the transmembrane domains (TMs) of zfGPCR and hGLP-1Rs with the hGCGR

Crystal structures of the 7TM domains of hGCGR [30] (PDB file 4L6R) and hCRF-R1 [31] (PDB entry 4K5Y) provided the first information about the interactions between different transmembrane helices (TMs) in the class B GPCRs. Mapping of the TMs in the zfGPCR and hGLP-1R, especially at the beginning of TM1 was based on the amino acid positions obtained from the 7TM crystal structure of hGCGR, which differs slightly from the predicted start from the SwissProt database used previously [55] [56]. The numbering of amino acids in the 7TM sequences follows the numbering system in the 7TM crystal structure of hGCGR [30] (PDB file 4L6R).

**Fig 6:**
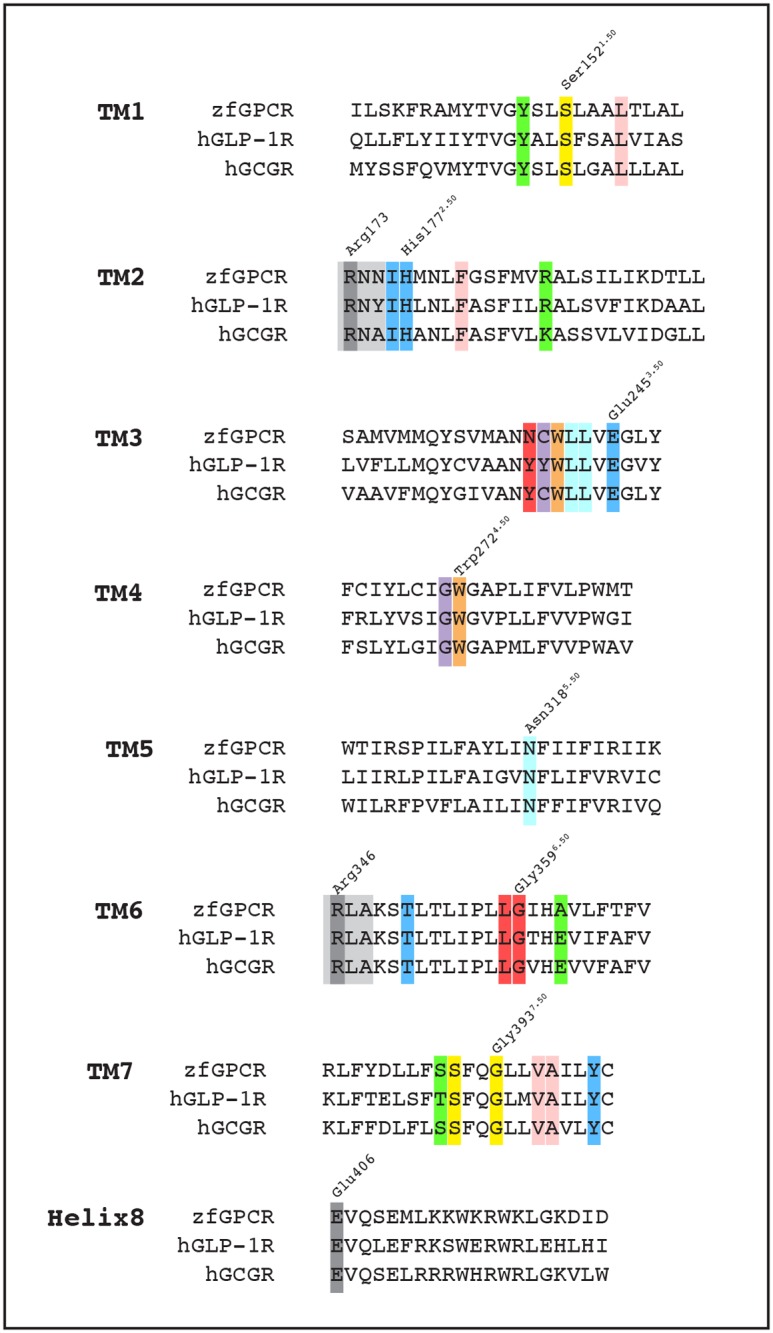
Multiple sequence alignment of TM helices and cytoplasmic helix 8 in zfGPCR, hGLP-1R and hGCGR. The most conserved residues in each TM are labeled using the modified Ballesteros-Weinstein generic numbering system [42, 43] (see Materials and Methods) and numbered according to the 7TM crystal structure of hGCGR [30] (PDB entry 4L6R). Conserved interactions between residues in different TM helices are highlighted in similar colors. The hydrogen bond network coordinated by Glu245^3.50^ (see Fig 7) is shown in dark blue. Residues outside of the TM domains are shaded light grey to highlight the conserved Arg residue that interacts with Glu406 (superscript numbers) in cytoplasmic helix 8 and are labeled and shaded dark grey.

Sequence alignments show that almost all of the amino acids identified in the 7TM crystal structures of hGCGR engaged in stabilizing the receptor's TM fold and in the interactions between different pairs of transmembrane helices are conserved in the corresponding positions in the transmembrane domains of zfGPCR and hGLP-1Rs (Fig 6) [30] (PDB file 4L6R). They are:

1. The two cysteine residues that form the disulfide bond between the extracellular loop 2 (ECL2) and TM3 (Fig 2, residues 294 and 224 ^3.39^, respectively).
2. Ser152^1.50^ in TM1 whose side chain forms a hydrogen bond with the backbone of Ser390^7.47^ in TM7 (conserved in hGLP-1R and corresponding to Ser392^7.47^). That same interaction is seen in the 7TM crystal structure of hCRF-R1 [31]: Ser152^1.50^ and Ser390^7.47^ correspond to Ser130^1.50^ and Ser 353^7.47^ in the hCRF-R1, respectively [31]. This interaction was also predicted by the modeling experiments of hGLP-1R [42].
3. Gly393^7.50^ that induces a bend in TM7 in hGCGR. In the 7TM crystal structure of hCRF-R1, a sharp kink is seen around the same glycine (in position 356^7.50^ in the hCRF-R1 sequence) that tilts the extracellular portion of TM7 outwards away from the helical bundle [31]. This Gly393^7.50^ is found in corresponding positions in the sequences of all class B GPCRs, including zfGPCR and it has been suggested that it allows flexibility important for the correct folding of class B GPCRs [57].
  In the 7TM crystal structure of hGCGR, the backbone nitrogen of Gly393^7.50^ forms a hydrogen bond with the oxygen of Ser152^1.50^. It is the same oxygen in Ser152^1.50^ that forms the hydrogen bond with the backbone of Ser390^7.47^ described above in (ii), and therefore likely contributes to the kink that is formed by Gly393^7.50^.
4. Phe181^2.54^ in TM2 that makes hydrophobic contacts with Leu156^1.54^ in TM1, Val396^7.53^ and Ala397^7.54^ in TM7 to stabilize TM1-TM2-TM7 interactions.
5. Trp272^4.50^ in TM4 whose side chain interacts with the side chain of Trp241^3.46^ in TM3.

The conservation of all these amino acids in zfGPCR and hGLP-1R (Fig 6) suggest that the same interactions likely exist in all three class B GPCRs.

**Fig 7:**
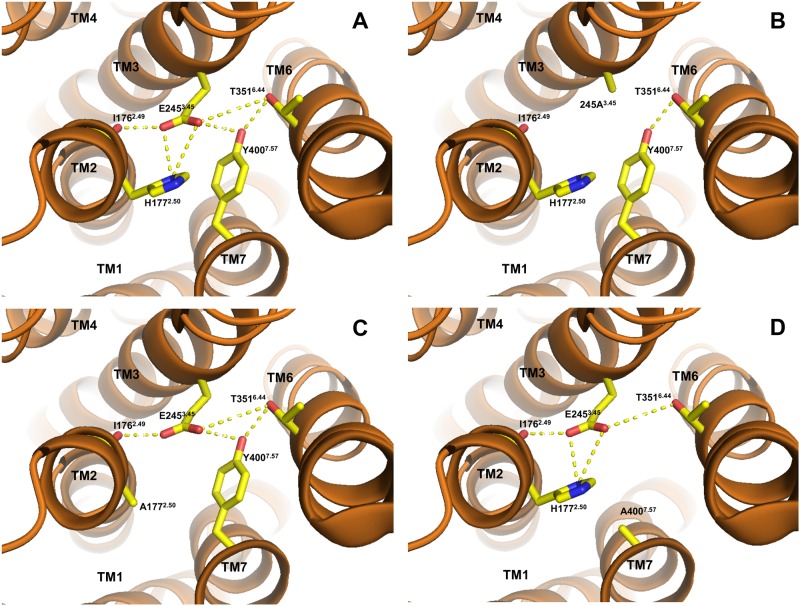
The TM2-TM3-TM6-TM7 hydrogen bond network coordinated by Glu245^3.50^ in TM3 with residues in TM6, TM2 and TM7 may contribute to the stability of the protein and thereby affect cell surface expression of hGCGR, zfGPCR and hGLP-1R (the contacts seen are those on the hGCGR, PDB entry 4L6R). In hGLP-1R, the mutation to alanine of Glu245^3.50^ in TM3 (Panel B), of His177^2.50^ in TM2 (Panel C) and of Tyr400^7.57^ in TM7 (Panel D) significantly reduced cell surface expression of GLP-1R [42]. Binding of ^125^I-exendin(9–39) to hGLP-1R could not be measured in these hGLP-1R mutants [42]. Alanine in these positions (Panels B, C, D) would not maintain the hydrogen bond network between all four transmembrane helices seen in the 7TM crystal structure of hGCGR.

The 7TM crystal structure of hGCGR also shows several interactions between TM helices that are maintained by contacts between amino acids shown by mutational analysis to be important in maintaining the correct structural fold and the cell surface expression of hGLP-1R [42]. This suggests that these same contacts may be important for the cell surface expression of hGCGR. Among them is the extensive hydrogen bond network between TM3-TM2-TM6-TM7 helices coordinated by Glu245^3.50^ in TM3 (Fig 7) that is conserved in zfGPCR and hGLP-1R (Fig 6). Glu245^3.50^ connects TM3 with TM2 through a hydrogen bond interaction with His177^2.50^, which is also conserved in zfGPCR and hGLP-1R (Fig 7, panel A, left). The same contact is seen in the 7TM crystal structure of hCRF-R1 between residues 155^2.50^ and 209^3.50^ corresponding to His177^2.50^ and Glu245^3.50^, respectively [31]. In addition, Glu245^3.50^ interacts through a hydrogen bond with the backbone oxygen of Ile176^2.49^ in TM2 (Fig 7, panel A, left).

In the TM6-TM3 interface the backbone of Glu245^3.50^ interacts with the side chain of Thr351^6.42^ in TM6, found in corresponding positions in the zfGPCR and hGLP-1R (Fig 7, panel A, right). Glu245^3.50^ makes another hydrogen bond with Tyr400^7.57^ in TM7, also conserved in zfGPCR and hGLP-1R (Fig 7, panel A, center).

Mutations to alanine in the hGLP-1R of residues Glu245^3.50^ in TM3, His177^2.50^ in TM2 and Tyr400^7.57^ in TM7 significantly reduced the cell surface expression of these hGLP-1R mutants [42]. Their surface expression was so low that no detectable binding of ^125^I-exendin(9–39) to the mutant hGLP-1Rs could be measured [42]. As seen from Fig 7, panels B, C, D, an alanine in positions 245^3.50^, 177^2.50^ and 400^7.57^ in hGCGR cannot form the same hydrogen bond interactions between TM3-TM2, TM3-TM7 and TM3-TM6 helices in hGCGR and could therefore disrupt the overall structure of the receptor.

This hydrogen bond network can also exist in zfGPCR and may also be important for its cell surface expression.

**Fig 8:**
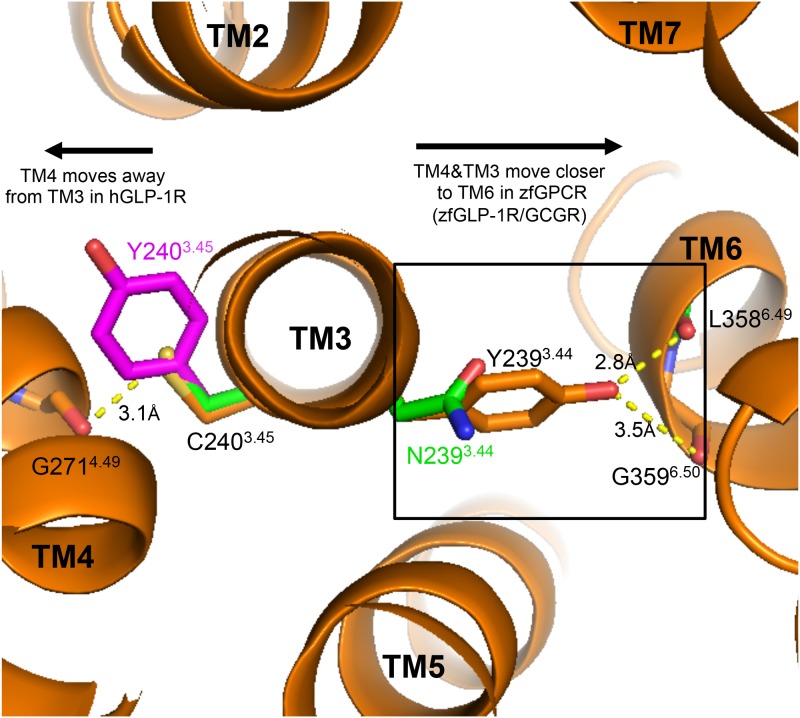
The predicted TM4-TM3-TM6 helical bundle would be more compact in zfGCGR than in hGGCR while in hGLP-1R is likely the most expanded. Cys240^3.45^ in TM3 of hGCGR [30] (PDB entry 4L6R; brown) forms side chain-to-backbone interaction with Gly271^4.49^ in TM4. Substitution of this cysteine with a tyrosine in hGLP-1R (magenta) should shift TM4 away from TM3 and TM6. TM3-TM6 interactions (box) of hGCGR between Tyr239^3.44^ in TM3 and main chain atoms of Gly359^6.50^ and Leu358^6.49^ in TM6 will be maintained in hGLP-1R, which has the same tyrosine, but not in zfGPCR with an asparagine (green) at this position. The shorter asparagine side chain should bring TM6 closer to TM3 in order to maintain these interactions.

Structural alignments between hGCGR and zfGPCR and hGLP-1R (Fig 8) show that the relative compactness of TM4-TM3-TM6 helices in these receptors is modulated by the composition of amino acids involved in the contacts between these helices. As seen in Fig 8 (box) interactions between TM3 and TM6 may be affected by the size of the residue side chains involved. In the 7TM crystal structure of hGCGR [30], the side chain of Tyr239^3.44^ in TM3 interacts with the backbone atoms of Gly359^6.50^ (3.5Å) and Leu358^6.49^ (2.8Å) in TM6. Tyr239^3.44^ is conserved only in hGLP-1R and is substituted with Asn239^3.44^ in the zfGPCR (Fig 6 red color and Fig 8). In order to make the same hydrogen bonding with the backbone of Gly359^6.50^ and Leu358^6.49^, the shorter side chain of Asn239^3.44^ may bring TM3 closer to TM6 in zfGPCR compared to hGLP-1R and hGCGR (Fig 8, box). The same analysis of side-chain size occurs in the TM3-TM4 interactions. In hGCGR, Cys240^3.45^ in TM3 forms a side chain-to-backbone interaction with Gly271^4.49^ (3.1Å) in TM4. In zfGPCR, this Cys240^3.45^ is conserved and therefore the same distance between TM3 and TM4 will be maintained in zfGPCR as in hGCGR (Fig 8, left side). But in hGLP-1R, the cysteine is replaced by Tyr240^3.45^ that would shift TM4 away from TM3 by some 4Å (Fig 8, left side). As a result, the overall configuration is that the TM4-TM3-TM6 helical bundle is likely more compact in zfGPCR and hGCGR than in hGLP-1R. Mutation of Cys240^3.45^ to Tyr240^3.45^ in hGCGR did not have an effect on its cell surface expression nor on its binding to glucagon [30] indicating that this TM3-TM4 interaction coordinated by Cys240^3.45^ in hGCGR or Tyr240^3.45^ in hGLP-1R may not be important either for the cell surface expression of these receptors nor binding to their ligands, but instead may contribute to the movements of their TM4-TM3 helices that would facilitate conformational changes in these receptors upon ligand binding.

Our inspection of the 7TM crystal structure of hGCGR [30] also identified a hydrophilic cluster formed by a salt bridge between Lys187^2.60^ in TM2 and Glu362^6.53^ in TM6, and hydrophilic portions of residues Tyr149^1.47^ in TM1, Gln232^2.38^, Asp238^2.43^, Tyr239^2.44^ in TM2; His361^6.52^ in TM6, and Asp385^7.42^, Ser389^7.46^ Gln392^7.49^ in TM7. This TM7-TM6-TM2-TM1 cluster offers a hydrophilic environment embedded deep inside the hydrophobic transmembrane. Mutational analysis of some of these residues in the hGCGR showed their importance in glucagon binding and led to a suggestion that the N-terminal end of glucagon extends deep into this pocket [30]. These residues in zfGPCR are conserved or are conservative substitutions: Lys187^2.60^ to arginine in zfGPCR and hGLP-1R (Fig 6) and Tyr239^2.44^ to asparagine in zfGPCR, except for Glu362^6.53^ that is an alanine in zfGPCR losing the salt bridge with the counterpart Arg187^2.60^. Mutation of Glu362^6.53^ to alanine in hGCGR led to a decrease in the binding of glucagon to the hGCGR and reduced surface expression of the mutated receptor [30]. Similar results were obtained by mutating the corresponding Glu362^6.53^ in hGLP-1R to alanine [55].

The intracellular helix 8, consisting of 20 amino acids and starting at position Glu406, seen in the 7TM crystal structure of hGCGR [30], shows a high degree of conservation between hGCGR, zfGPCR and hGLP-1R (Fig 6, grey color). Glu406 is fully conserved among all the class B GPCRs and forms two interhelical salt bridges with Arg173^2.46^ and Arg346^6.37^, which are also conserved in zfGPCR and hGLP-1R.

Interactions between different TM helices are summarized in Table 1.

**Table 1:** Summary of the sequence and structural mapping of the 7TM domains in zfGPCR and hGLP-1R onto hGCGR based on the 7TM structure of hGCGR ([30].

CONSERVED STRUCTURAL ELEMENTS IN 7TMs			
hGLP-1R	hGCGR	zfGPCR	Shown in figures
Conserved	Disulfide bond	Conserved	Fig 2, yellow lines
Conserved	TM1-TM7 side chain to backbone interaction	Conserved	Fig 6, yellow color
Conserved	Gly 393 in TM7 important for correct folding of class B GPCRs	Conserved	Fig 6, yellow color
Conserved	TM1-TM2-TM7	Conserved	Fig 6, pink color
Conserved	TM4-TM3	Conserved	Fig 6, orange color
Conserved	TM3-TM2-TM6-TM7 hydrogen bond network coordinated by Glu245 in TM3	Conserved	Fig 6, dark blue color; Fig 7(A)–7(D)
Conserved	TM5-TM3 side chain to back bone interaction	Conserved	Fig 6, light blue color
Change in TM4-TM3 compactness	TM4-TM3-TM6	Change in TM3-TM6 compactness	Fig 6, red color; Fig 8
Conserved	TM7-TM6-TM2-TM1 hydrophilic cluster	Hydrophilic cluster conserved but not all residues	Fig 6, green color
Conserved	Helix 8 not in 7TM	Conserved	Fig 6, dark grey color

### Mapping of the structural features of the NECD of the hGCGR to the NECD sequences of zfGPCR and hGLP-1R

Structural and sequence mapping of the 7TM of zfGPCR with the corresponding region in the hGCGR based on the 7TM crystal structure of hGCGR [30] showed the likely conservation of many structural features in the 7TM helical bundle in the zfGPCR (Table 1, Figs 6, 7 and 8). These observations suggested that we should extend the sequence and structural mapping of the zfGPCR to include the NECD of hGCGR (Fig 3), using as a reference structure the crystal structure of the NECD of hGCGR in complex with Fab fragments of several monoclonal antibodies that block the binding of human glucagon and inhibit the basal receptor activity [32] (PDB entry 4ERS). Although there is little sequence conservation between the two receptors in the region 32 to 52 (Fig 3), the secondary structure calculations shows the conservation of the amphiphilic helix in this region of zfGPCR and hGCGR (S1 Fig).

The crystal structure (PDB entry 4ERS) identified several residues to be critical for the integrity of the NECD of hGCGR [32]. To be able to correlate the structural features identified in the NECD of hGCGR with those of zfGPCR and hGLP-1R described earlier, the numbering of amino acids in the NECD of hGCGR follows the numbering of the NECD of hGLP-1R shown in Fig 3. The crystal structure shows that Asp67, Lys102, Arg121, Trp72 and backbone amide of Ser70 (Fig 3) play an important role in maintaining the structural integrity of the NECD of hGCGR by forming a salt bridge between Asp67, Lys102 and Arg121 and hydrogen-bonds with Trp72 and the backbone amide of Ser70. These interactions are equivalent to the hydrogen bond network that involves Asp67, Arg102, Arg121 and Trp72 observed in the hGLP-1R NECD [35, 36] and shown in Fig 5 for hGLP-1R and zfGPCR. The similarity of this hydrogen bond network in the two receptors implicates an interaction between Arg121 and the main chain atom at position 27 of glucagon, i.e. methionine (Fig 1), analogous to the interactions between Arg121 and amino acids in position 27 of exendin(9–39) and hGLP-1 identified in the crystal structures of the hGLP-1R NECD (Fig 5). This interaction is also predicted by the Koth *et al*. model [32]. In addition, the crystal structure identified Tyr68 and side chains of Trp72 and Trp110 as residues that form a core of the NECD of hGCGR and are conserved in hGLP-1R NECD and zfGPCR.

All residues involved in structural stability mentioned above are conserved in zfGPCR with the exception of Lys102 in hGCGR that is an arginine in both hGLP-1R and zfGPCR and Arg 121 is a lysine in zfGPCR (Fig 3), substitutions that maintain the salt bridge integrity (Fig 5). Ser70 is substituted with valine, but this should not affect the structural integrity of the zfGPCR because interaction with Val70 is a backbone interaction.

A model for the interaction of glucagon with hGCGR [32] suggested the discontinuous segment of other basic amino acids, Lys68 (64 in hGCGR), Lys102 (98 in hGCGR), Arg112 (108 in hGCGR), Arg115 (111 in hGCGR), Gln117 (113 in hGCGR) and Arg121(116 in hGCGR) to be important for glucagon binding to hGCGR. Substitutions in these positions in hGLP-1R (and zfGPCR) change the nature of the charge in this segment of these receptors and therefore hypothesized by Koth et al [32]to be responsible for the ligand specificity of hGLP-1R and hGCGR. Three of these residues are in the loop 3 of hGCGR and hGLP-1R. As described earlier, loop 3 is absent in the zfGPCR (Figs 3 and 4). The sequence of loop 3 in hGCGR is shorter by one amino acid compared to hGLP-1R (Fig 3). In the hGCGR there is an unusual turn in the sequence Gly109-Gly112 not seen either in the crystal structure of the NECD of hGLP-1R in complex with exendin(9–39) [35] nor in complex with hGLP-1.

Results obtained from the sequence and structural mapping of the NECD of zfGPR onto the structural features of the NECD of hGLP-1R and hGCGR based on the crystal structures of their NECDs [32, 35, 36] are summarized in Table 2.

**Table 2:** Summary of the sequence and structural mapping of the NECD of zfGPCR onto the NECDs of GLP-1R and hGCGR based on their crystal structures [32, 35, 36].

CONSERVED STRUCTURAL ELEMENTS IN NECDs			
hGLP-1R	hGCGR	zfGPCR	Shown in figures
Residues forming 3 disulfide bonds	Conserved	Conserved	Fig 2, yellow lines Fig 3, orange color
Residues stabilizing the core of class B GPCRs	Conserved	Conserved	Fig 3, blue color
Residues that play a role in the intramolecular interactions in class B GPCRs ^(a)^	Conserved	Conserved	Fig 3
Cluster of Trp residues	Conserved	Conserved	Fig 3, an asterisk below the alignment
Amphiphilic helix between residues 32–52	Conserved	Conserved	Fig 3, grey shading; S1 Fig
Aromatic hydrophobic cluster	Most residues forming the aromatic cluster not conserved	Aliphatic hydrophobic cluster	Figs 3 and 4
Loop 3	Conserved	Absent	Fig 3, pink color; Fig 4
Trp 120	Conserved	Gly 120	Figs 3 and 4
Hydrogen bond network coordinated by Asp 67	Similar ^(b)^	Conserved	Fig 3, residues marked with hash sign; Fig 5
Interaction of Arg 121 with residue 27 of GLP-1 and exendin(9–39)	Similar interaction of Arg 121 with Met 27 in glucagon^(c)^	Conserved, but Arg 121 is substituted with Lys 121 that interacts with residue 27 of GLP-1 and exendin(9–39)	Fig 5
Residues important for forming the exendin(9–39) hydrophobic binding pocket	Not analyzed	Conserved	Fig 3, yellow color; and blue and yellow hatching

^(a)^ Described in the Results under “Mapping of the structural features of the NECD of hGLP-1R onto the sequence of the NECD of zfGPCR”and in Discussion under “Role of the NECD”

^(b)^ Described in the section “Mapping of the structural features of the NECD of the hGCGR to the NECD sequences of zfGPCR and hGLP-1R according to Koth et al. [32]

^(c)^ Described in the model by Koth et al [32]

### Functional characterization of the zfGPCR

Results summarized in Tables 1 and 2 showed considerable structural conservation between zfGPCR, hGLP-1R and hGCGR and suggested that we should extend our initial characterization of the ligand specificity of the zfGPCR towards zfGLP-1, hGLP-1 and exendin-4 to include ligand specificity of this receptor towards glucagon sequences.

#### (A). Ligand binding specificity of the zfGPCR towards zebrafish and human glucagons measured in competitive binding experiments using ^125^I-hGLP-1(7–36)amide as tracer

**Fig 9:**
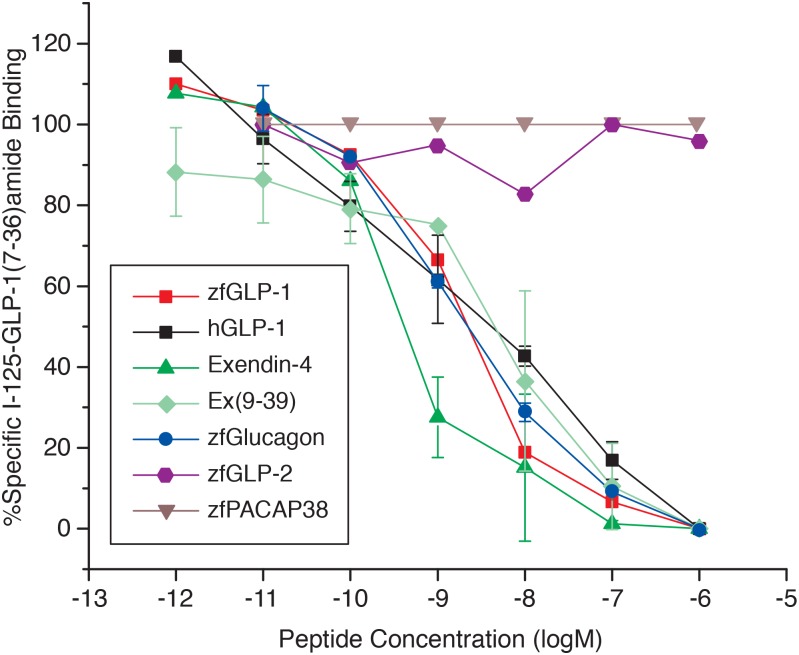
Zebrafish glucagon displaces the binding of radioiodinated ^125^I-hGLP-1(7–36)amide to the recombinant zfGPCR in a similar dose-dependent manner as zfGLP-1, hGLP-1, exendin-4 and exendin(9–39). Displacement curves represent average measurements obtained from three separate rounds of transfections for the displacement with hGLP-1 (n = 3), two for exendin-4, exendin(9–39) and zebrafish glucagon (n = 2) and one for zfGLP-1, zfGLP-2 and zebrafish PACAP-38 (n = 1). Data points for each concentration in each displacement curve obtained in a single round of transfection are an average of three independent measurements. Error bars are shown for data points in the displacement curves obtained in two or more rounds of transfections (see Materials and Methods).

We used zebrafish glucagon, zfGLP-1 and hGLP-1, exendin-4 and exendin(9–39) to displace the binding of ^125^I-hGLP-1(7–36)amide to the recombinant zfGPCR expressed transiently in COS-7 cells. For comparison in these experiments, we also included zebrafish GLP-2 and zebrafish PACAP-38 (for sequences see Fig 1). As seen in Fig 9 with the exception of zebrafish GLP-2 and zebrafish PACAP-38, all peptides displaced the ^125^I-GLP-1(7–36) amide binding in a dose-dependent manner with similar IC_50_s (Table 3, Panel A). The strongest binding, as measured by IC_50_, was observed with exendin-4 (0.48nM, 95% CI: 0.24nM to 0.92nM), in agreement with our earlier finding [41]. Remarkably, the IC_50_ for zebrafish glucagon (2.4 nM, 95% CI: 1.4 nM to 4 nM) was similar to the IC_50_s for zfGLP-1 (2.1nM, 95% CI: 1.2 nM to 3.6 nM), hGLP-1 (3.6 nM, 95% CI: 0.98 nM to 13 nM), and exendin(9–39) (5.8 nM, 95% CI: 2.4 nM to 14 nM) (Table 3, panel A).

**Table 3:** ZfGPCR does not significantly discriminate between zfGLP-1, hGLP-1, zebrafish glucagon and human glucagon, as determined from competitive binding experiments shown in Figs 9 and 10.

	(A) ^125^I-hGLP-1(7–36)amide binding			(B) ^125^I- exendin(9–39) binding		
Peptide in displacement curve	IC_50_(nM)^a^	95% Confidence interval (nM)^b^	Difference from zfGLP-1 IC_50_ (P values)^c^	IC_50_ (nM)^e^	95% Confidence interval (nM)^b^	Difference from zfGLP-1 IC_50_ (P values)^c^
zfGLP-1	2.1	1.2–3.6	control	290	225–371	control
hGLP-1	3.6	0.98–13	0.3286	178	121–262	0.0311^d^
zf glucagon	2.4	1.4–4.0	0.6766	106	78.5–144	<0.0001^d^
h glucagon	N.D.	N.D.	N.D.	67	33.6–134	0.0009^d^
exendin-4	0.48	0.24–0.92	0.0014^d^	1.00	0.84–1.17	<0.0001^d^
exendin(9–39)	5.8	2.4–14	0.0543	4.21	3.42–5.17	<0.0001^d^

^(a)^ IC_50_ values were calculated with Prism 4 software from the displacement curves shown in Fig 9 when ^125^I-hGLP-1(7–36)amide was used as a radioactive tracer and represents the concentration of peptide that inhibited the specific binding by 50%.

^(b)^ Calculated by the Prism 4 software.

^(c)^ Difference between the displacement curves for zfGLP-1 and other peptides used in the competitive binding experiments were calculated using the F-test in the Prism 4 software that compares the fitted mid-point (log IC_50_) of the zfGLP-1 displacement curve with the fitted midpoints (log IC_50_) for the other peptides.

^(d)^ P<0.05 is statistically significant.

^(e)^ IC_50_ values were calculated with Prism 4 software from the displacement curves shown in Fig 10 when ^125^I-exendin(9–39) was used as a radioactive tracer and represent the concentration of peptides that inhibited the specific binding by 50%.

#### (B). Ligand binding specificity of the zfGPCR towards zebrafish and human glucagons measured in competitive binding experiments using ^125^I-exendin(9–39) as tracer

**Fig 10:**
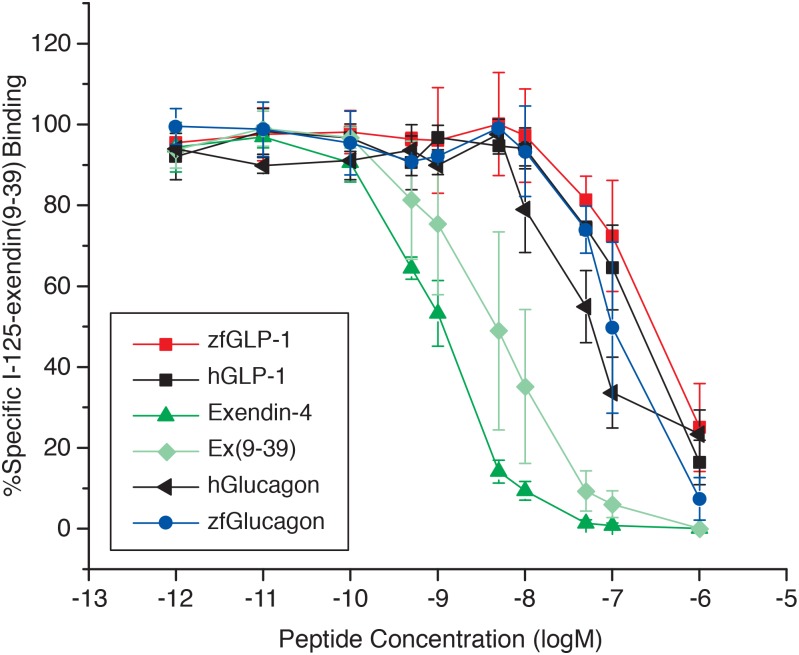
Higher concentrations of zfGLP-1, hGLP-1, zebrafish glucagon and human glucagon are required to displace 50% of the binding of ^125^I-exendin(9–39) to the recombinant zfGPCR relative to exendin-4 and exendin(9–39). Compare to Fig 9. Displacement curves with zfGLP-1 represent average measurements obtained from four separate rounds of transfections (n = 4), for hGLP-1, zebrafish glucagon, human glucagon and exendin-4 from three (n = 3) and for exendin(9–39) from five (n = 5). Each data point in the displacement curve obtained in a single round of transfection is an average of three independent measurements.

We further used zebrafish glucagon, human glucagon, zfGLP-1, hGLP-1, exendin(9–39) and exendin-4 to displace the binding of ^125^I-exendin(9–39) to the recombinant zfGPCR (Fig 10 and Table 3, Panel B). Exendin(9–39) displaced the binding of its radioiodinated peptide with a similar IC_50_ as the one determined when ^125^I-hGLP-1(7–36) amide was used as tracer (4.2 nM, 95% CI: 3.4 nM to 5.2 nM vs 5.8 nM, 95% CI: 2.4 nM to 14 nM, respectively) (Table 3, Panels A and B). The IC_50_ for exendin-4 (1nM, 95% CI: 0.8 nM to 1.2 nM) determined from the displacement curve with ^125^I-exendin(9–39) binding was similar to the value obtained from a displacement curve with ^125^I-hGLP-1(7–36)amide binding (0.48 nM, 95% CI: 0.24 nM to 0.92 nM, p = 0.0056) to the recombinant zfGPCR (Table 3, Panels A and B).

But the IC_50_’s obtained from dose-dependent displacement of ^125^I -exendin(9–39) binding with zfGLP-1and hGLP-1 were about 50–150 fold higher than IC_50_’s obtained from the dose-dependent displacement of ^125^I-hGLP-1(7–36)amide binding with these peptides, i.e 290 nM vs 2.1 nM for zfGLP-1 and 178 nM vs 3.6 nM for hGLP-1 (Table 3, Panels A and B). Comparable differences in IC_50_’s were observed when ^125^I-exendin(9–39) binding was displaced with zf glucagon (106 nM vs 2.4 nM). Human glucagon displaced the binding of ^125^I-exendin(9–39) with an IC_50_ of 67 nM (95% CI: 33.6 nM to 134 nM) (Table 3, Panel B). The IC_50_’s for hGLP-1, zfglucagon and human glucagon obtained from the dose- dependent displacement of ^125^I-exendin(9–39) binding to zfGPCR were significantly different (p = 0.0311, p< 0.0001, p = 0009, respectively) from IC_50_ for zfGLP-1 (Table 3, panel B). This is in contrast to the IC_50_’s obtained from the competitive binding experiments when ^125^I-hGLP-1(7–36)amide was used a as tracer (Table 3, Panel A).

#### Intracellular cAMP levels in COS-7 cells expressing the recombinant zfGPCR

**Fig 11:**
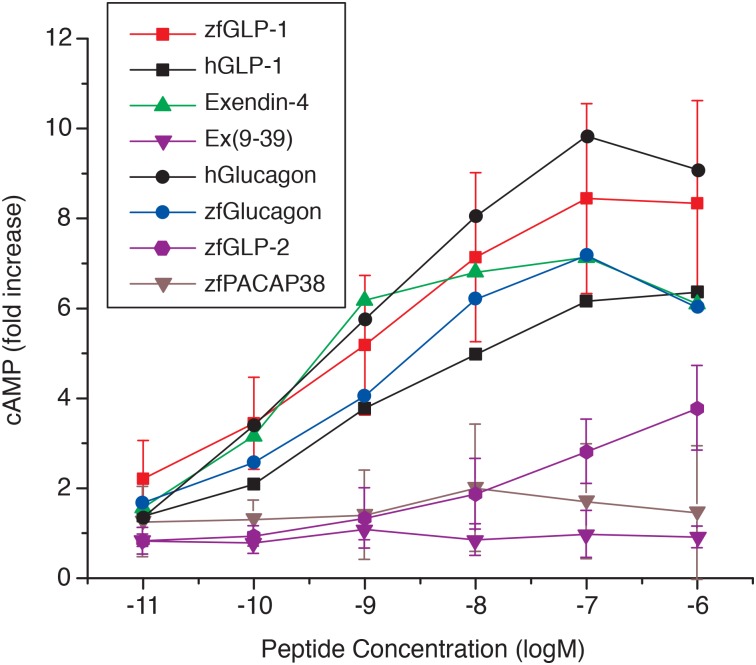
ZfGLP-1 (n = 9), hGLP-1 (n = 5), zebrafish glucagon (n = 5), human glucagon (n = 5) and exendin-4 (n = 3) stimulate intracellular cAMP through the recombinant zfGPCR in a similar dose-dependent manner. Exendin(9–39) (n = 4) and zebrafish PACAP-38 (n = 2) have no effect, and zfGLP-2 (n = 6) has stimulatory effects only at much higher concentrations than the other tested peptides that stimulated cAMP. (n) represents number of separate rounds of transfections. Each data point in the dose-response curve obtained in a single transfection is an average of three separate measurements (see Materials and Methods). To highlight differences between stimulatory effects of zfGLP-1, zebrafish glucagon, human GLP-1, human glucagon and exendin-4 from the stimulatory effect of zfGLP-2 error bars are shown only for zfGLP-1, zfGLP-2, exendin(9–39) and zfPACAP-38.

Functional responses of the zfGPCR elicited after binding of zebrafish glucagon and human glucagon were determined by measuring the stimulation of intracellular cAMP levels. For comparison, zfGLP-1 and hGLP-1, exendin-4, exendin(9–39), zfGLP-2 and zebrafish PACAP-38 were also used. As seen from Fig 11 and Table 4, zebrafish glucagon and human glucagon increased cAMP levels in a dose-dependent manner and with EC_50_s in nM concentrations (0.76nM, 95% CI: 0.2nM to 2.9nM, and 0.54nM, 95% CI: 0.18nM to 1.63nM, respectively) similar to zfGLP-1 (0.44nM, 95% CI: 0.24nM to 0.8nM), hGLP-1 (1nM, 95%: 0.65nM to 1.63nM) and exendin-4 (0.18nM, 95% CI: 0.07nM to 0.46nM). The maximum stimulatory effects with all tested peptides with the exception of zfGLP-2 were observed at concentrations of 0.1μM.

**Table 4:** Zebrafish glucagon and human glucagon stimulate intracellular cAMP mediated through the recombinant zfGPCR to a similar degree as zfGLP-1 and hGLP-1, as determined from the dose-response curves shown on Fig 11.

Peptide ligand	EC_50_ (nM)^a^	95% confidence interval (nM)^b^	Difference from zfGLP-1 EC_50_ (P value)^c^
zf GLP-1	0.44	0.24–0.80	control
h GLP-1	1.00	0.65–1.63	0.0138^d^
zf glucagon	0.76	0.20–2.9	0.3438
hglucagon	0.54	0.18–1.63	0.6796
exendin-4	0.18	0.07–0.46	0.0861
zf GLP-2	35	16.8–72.6	P<0.0001^d^

^(a)^ EC_50_ values represent peptide concentration that produced 50% of the maximum intracellular cAMP concentration and were calculated by Prism 4 software using a four-parameter logistic sigmoidal curve fit.

^(b)^ Calculated by Prism 4 software.

^(c)^ Differences between zfGLP-1 cAMP dose-response curve and the dose-response curves for other peptides were calculated using the F-test that compares the fitted mid-points (logEC_50_).

^(d)^ P<0.05 is statistically significant

The hGLP-1R antagonist, exendin(9–39), did not show any stimulatory effects on cAMP levels even at a high concentration of 1μM, and thus behaved as an antagonist of the zfGPCR analogous to rGLP-1R and hGLP-1R [23, 24]. ZfGLP-2 was a weak agonist of the zfGPCR with an EC_50_ of 35 nM (95% CI: 16.8 nM to 72.6 nM) and had a cAMP dose response curve distinct from that of the other peptides tested in this assay. Zf PACAP-38 did not stimulate cAMP at all tested concentrations. Taken together, the results of our functional experiments indicate that exendin-4, zfGLP-1, hGLP-1, zebrafish glucagon and human glucagon stimulate intracellular cAMP levels mediated by the zfGPCR to a similar extent, but their binding to the receptor may stabilize different conformational states of the receptor.

Our functional experiments demonstrate that we need to modify our previous conclusion that we had isolated a GLP-1R in zebrafish. Instead, we have characterized a G-protein coupled receptor in zebrafish with dual ligand specificity towards both GLP-1 and glucagon. This type of receptor is not found in mammals.

## Discussion

### Ligand specificity of the dual zebrafish GLP-1R/GCGR

This study demonstrates that the zfGPCR which we characterized previously [41] and in this study combines the ligand binding specificity (Figs 9 and 10, Table 3) and cAMP responses (Fig 11, Table 4) of hGLP-1R [23] and glucagon receptors in fish, frogs and mammals [18, 19] [13] [58]. We therefore are calling this zebrafish receptor a dual zfGLP-1R/GCGR so as to characterize its multi-ligand recognition.

To be consistent with our initial characterization of this receptor which we referred to as zfGLP-1R [41] and the characterization of the gfGCGR [18] we used only the sequences of zfGLP-1 and zf glucagon used in those studies (shown in Fig 1) and not the second zfGLP-1 and second zebrafish glucagon found in the zebrafish genome. In the competitive binding experiments using^125^I-hGLP-1(7–36)amide as tracer IC_50_ ‘s for zfGLP-1 and hGLP-1 which sequences differ in 10 out of 31 amino acid were within similar concentration range (Table 3, Panel A), i.e. 2.1 nM (CI:1.2 nM -3.6 nM) and 3.6 nM (CI: 0.98 nM -13 nM), respectively. These results suggest that zfGLP-1R/GCGR will likely bind the closely related second zfGLP-1. In the same competitive binding experiments with zfGLP-1R/GCGR IC_50_ for zebrafish glucagon (2.4nM, 95% CI: 1.4nM to 4.0nM) (Table 3, Panel A) is similar to the IC_50_’s for goldfish, zebrafish and human glucagons previously determined from displacements curves with other vertebrate GCGRs. Thus, in the competitive binding experiments with the recombinant glucagon receptor from the goldfish *C*. *auratus* the IC_50_ ‘s for zebrafish glucagon (which sequence corresponds to zebrafish glucagon a, Gcga [51], also referred to as zebrafish 1 glucagon [59]) and goldfish glucagon were 8.7nM (95% CI: 4.3nM-18nM) and 0.56nM (95% CI: 0.31nM to 1nM), respectively [18].

In addition the IC_50_ for human glucagon determined in the competitive binding assays with the recombinant glucagon receptor from the frog *R*. *tigrina rugulosa* was 12nM [19] versus 5nM for the recombinant hGCGR [58].

EC_50_’ s determined from the stimulation of intracellular cAMP levels by zebrafish and human glucagons (0.76 nM, 95% CI: 0.2nM to 2.9nM; and 0.54 nM, 95% CI: 018nM to 1.63nM, respectively) (Table 4) were similar to the recently reported EC_50_ values obtained for cAMP stimulations by zebrafish glucagon a (Gcga) (4.16 nM, 95% CI: 1.25 nM to 15.38nM) and zebrafish glucagon b (Gcgb) (14.61nM, 95% CI: 5.827nM to 36.64 nM) mediated through the zebrafish Gcgra receptor [51] that shows 87% identity to the goldfish GCGR that we characterized previously [18]. In those studies the EC_50_ for goldfish glucagon and human glucagon were 3 nM (95% CI: 1.3 nM to 7 nM) and 9.7 nM (95% CI: 4.7nM to 20 nM), respectively. [18]

In addition, EC_50_’s for the stimulations with zebrafish glucagon a (Gcga) and zebrafish glucagon b (Gcgb) mediated through the zebrafish Gcgrb [51],which sequence corresponds to the zfGLP-1R/GCGR sequence characterized in this study, were 27.98 nM (95% CI: 13.54nM to 57.79nM) and 14.88 nM (95% CI: 8.770 nM to 25.24 nM), respectively.

Moreover, EC50’s for human and zebrafish glucagon obtained in our study were within similar concentration range as the EC_50_ ‘s determined in experiments with recombinant glucagon receptors from other vertebrate species. Thus, EC_50_‘s for human glucagon obtained from the cAMP assays performed with the recombinant glucagon receptor from the frog and the recombinant rat glucagon receptor were 0.8 nM [19] and 0.7nM [13], respectively, and are similar to the EC_50_ value of 1nM determined for the glucagon receptor in rat hepatocytes [60].

zfGLP-2, encoded by the second proglucagon gene in zebrafish [59], elicited cAMP responses starting at 10 nM concentrations and the maximum increase of cAMP obtained at 1μM peptide was several fold lower compared to that achieved with exendin-4, zfGLP-1, hGLP-1, zebrafish glucagon and human glucagon (Fig 11, Table 4). The discrepancy between the results obtained in the competitive binding and the cAMP experiments with zfGLP-2 (Fig 9, Table 4) was most likely due to different sensitivities of the two assays. Zebrafish PACAP-38, which belongs to the glucagon-secretin family of peptides and which binds to the zebrafish PACAP Type 1 and PACAP Type 2 receptors with IC_50_ ‘s of 7.5 nM and 6 nM, respectively [61], did not displace the binding of ^125^I-hGLP-1(7–36) amide to the zfGLP-1/GCGR even at high μM concentrations. It also did not stimulate intracellular cAMP levels, suggesting that the cAMP responses mediated through the zfGLP-1/GCGR are restricted to exendin-4 and peptides derived from preproglucagon.

We observed big differences between the IC_50_ values for zfGLP-1, hGLP-1, zf glucagon and human glucagon obtained in the competitive binding experiments when ^125^I-exendin(9–39) and ^125^I-hGLP-1(7–36)amide were used as tracers (Figs 9 and 10, Table 3, Panels A and B). In contrast, similar IC_50_’s for hGLP-1 were obtained from the competitive binding experiments with hGLP-1R when ^125^I-hGLP-1(7–36) amide and ^125^I-exendin (9–39) were used as tracers [42, 54, 55]. The large increase in IC_50_ values (about 50 to 150 fold) obtained from the dose dependent displacement of ^125^I- exendin(9–39) binding vs. ^125^I-hGLP-1(7–36)amide binding with zebrafish GLP-1 and hGLP-1 indicate that once exendin(9–39) is bound to zfGLP-1/GCGR the receptor conformation is less amenable to GLP-1 peptide binding. It is possible exendin(9–39) locks the zfGLP-1/GCGR into a non-productive conformation leading to poorer recognition of zfGLP-1 and hGLP-1. This analysis applies to the glucagon peptides as well (Figs 9 and 10, Table 3, Panels A and B). However, zfGLP-1, hGLP-1, zf glucagon and human glucagon induced similar dose-dependent increases in intracellular cAMP levels (Fig 11) suggesting that their binding to zfGLP-1R/GCGR leads to fully active receptor conformation(s).

Of all the peptides tested exendin-4 had the lowest IC_50_ ‘s of 0.48 nM and 1nM obtained from the dose-dependent displacement of ^125^I-hGLP-1 (7–36)amide and ^125^I-exendin(9–39) binding to zfGLP-1R/GCGR, respectively, compared to other peptides used (Figs 9 and 10, Table 3, Panels A and B). Lower IC_50_ for exendin-4 compared to the zfGLP-1R/GCGR antagonist exendin(9–39) (Figs 9 and 10, Table 3, Panels A and B) indicates that exendin-4 binds to the 7TM domain of zfGLP-1R/GCGR. It is likely that the contacts between His-1** and other N-terminal residues of exendin-4 and the 7TM domain of zfGLP-1R/GCGR lead to stronger binding of exendin-4 compared to exendin (9–39) which lacks the first eight N-terminal residues. Lower IC_50_ obtained for exendin-4 vs zfGLP-1, hGLP-1, zf glucagon and human glucagon likely results from a better alignment of its C-terminal end to the NECD of zfGLP-1R/GCGR compared to these peptides due to the predicted helical nature between residues 9 and 39 in exendin-4 [62]. Differences in IC_50_’s between exendin-4 and hGLP-1 obtained from the competitive binding experiments with zfGLP-1R/GCGR are similar to the results obtained with hGLP-1R and rGLP-1R when the binding of ^125^I-GLP-1(7–36) amide was displaced with exendin-4 and hGLP-1 [23, 41]. Despite the big differences between IC_50_ for exendin-4 and IC_50_’s for zfGLP-1, hGLP-1, zf glucagon and human glucagon (Table 3, Panels A and B) binding of exendin-4 to zfGLP-1R/GCGR did not lead to more potent receptor activation compared to other tested peptides with stimulatory cAMP effects, as measured by the EC_50_ values obtained from the cAMP dose-response curves (Fig 11, Table 4).

Taken together results from our functional experiments extend several previous findings and establish that what we referred to as zfGLP-1R [41] is not a zfGLP-1R and furthermore that zfGcgrb [51] also referred to as zfGCGR2 [50] is not a zfGCGR. Instead, these different names refer to a single receptor with dual ligand selectivity towards GLP-1 and glucagon and we call it a dual zfGLP-R/GCGR to highlight its dual ligand specificity towards both GLP-1 and glucagon.

Our functional studies also established that zfGLP-1R/GCGR has broad ligand specificity towards GLP-1 and glucagon sequences. Thus zfGLP-1R/GCGR has similar ligand specificity towards both zfGLP-1 and hGLP-1 which sequences differ in 10 out of 31 amino acids and towards both zf glucagon and human glucagon which sequences differ in 7 out of 29 amino acids (Fig 1). These findings suggest that zfGLP-1R/GCGR will likely have ligand specificity to the closely related second zfGLP-1 as well as the second zebrafish glucagon that differs only in the C-terminal amino acid from the sequence of zebrafish glucagon used in our experiments.

In our functional characterizations of GPCRs for GLP-1 and glucagon in zebrafish we did not focus on zfGCGR because our earlier extensive functional and phylogenetic characterization of gfGCGR [18] demonstrated that zfGCGR also referred to as Gcgra [51] and zfGCGR1 [50]is a homolog of gfGCGR. These conclusions were supported by the following observations: (i) gfGCGR shows 87% sequence identity to the zebrafish Gcgra/zfGCGR1 and groups by phylogentic analysis with the zebrafish Gcgra /zfGCGR1 [50, 51]; (ii) gfGCGR has ligand specificities towards both glucagon a (corresponding to the sequence of glucagon shown in Fig 1) and likely glucagon b, which sequence corresponds to gf glucagon with a single conservative amino acid substitution in position 24 [18]; (iii) as discussed earlier EC_50_ for glucagon a and goldfish glucagon obtained in measurements of cAMP responses mediated through the gfGCGR are in a good agreement with the values obtained for glucagon a and glucagon b mediated by Cgcra [51].

Most importantly, our functional results demonstrate that accurate assignments of ligands for GPCRs in zebrafish and other teleost fish that contain multiple related receptors for multiple related ligands [49] [50] require extensive characterizations that will correlate functional experiments with phylogenetic analysis and syntheny mapping. The dual ligand specificity of zfGLP-1R/GCGR towards GLP-1 and glucagon could not have been predicted from the phylogentic analysis. But, the functional characterization of the two GCGRs, i.e. gfGCGR [18] and zf GCGR [51] is in agreement with the phylogenetic analysis that grouped them with the other members of the vertebrate GCGRs [18] [49, 50] [51]. It is noteworthy that glucagon peptides found in different vertebrates are able to elicit functional responses from homologous gfGCGR and zfGCGR despite differences in their amino acid sequences [18] [51].

The broad ligand specificity of zfGLP-1R/GCGR towards other multiple GLP-1 and glucagon sequences found in other fish was not a focus of our studies and requires further investigation. Instead, we wanted to provide an expalanation for the dual ligand specificity of zfGLP-1R/GCGR and analyzed the structural features of the the dual zfGLP-1R/GCGR.

### Structural features of the dual zfGLP-1R/GCGR

Sequence and structural mapping of the zfGLP-1R/ GCGR show that it may contain similar structural features as the ones seen in the crystal structures of the NECD of hGLP-1R in complex with exendin(9–39) [35] and in complex with hGLP-1 [36]) as well as in the crystal structure of the NECD of hGCGR [32] (Table 2) and the 7TM crystal structure of hGCGR [30] (Table 1). Among the predicted conserved structural elements in the NECD and 7TM domains are: (i) the amphiphilic helix in its NECD between amino acids 31 and 48 (S1 Fig); (ii) side chain-to-backbone interactions that stabilize conformations important for ligand binding and cell surface expression [30] [42]; and (iii) several hydrogen bond networks important for ligand binding and structural integrity of the receptors (Figs 5 and 7) [35, 36], [30, 42].

### Role of the NECD

As shown in Fig 3 and Table 2 most of the residues that are important for stabilizing the core of class B GPCRs and play a role in the intramolecular interactions in the NECD are conserved in the zfGLP-1R/GCGR. Among them are several tryptophan residues. Most of them (Trp39, Trp72, Trp91 and Trp110) are essential for ligand binding directly or for structural integrity essential for ligand binding: mutation of any of them to Ala resulted in complete loss of binding to full-length rGLP-1 [52, 53]. But the substitution of Trp87 had no effect on binding or activation [53], suggesting that although Trp87 is engaged in intramolecular interaction with Tyr42 of the NECDs in hGLP-1R and likely in zfGLP-1/GCGR it has no direct role either in binding to GLP-1 or in receptor activation. The predicted amphiphilic helix in the NECD of the zfGLP-1R/GCGR (S1 Fig) may play a role in the initial alignment of the C-terminal ends of zfGLP-1, hGLP-1, exendin-4, exendin(9–39), zebrafish glucagon and human glucagon, analogous to the role of the amphiphilicity of the helices in hGLP-1R, [35, 36] and hGCGR [32], respectively (S1 Fig). Substitution of Leu32 at the beginning of the amphiphilic helix in hGLP-1R with Gln32 in the predicted amphiphilic helix in the zfGLP-1/GCGR did not significantly change the IC_50’_s obtained from the competitive binding experiments of zfGLP-1R/GCGR with exendin-4, exendin(9–39) and hGLP-1 (Fig 9, Table 3, Panel A) compared to the IC_50_ values obtained from the competitive binding experiments of rGLP-1R and hGLP-1R with these peptides [23, 41].

Despite low sequence identity within this helix, calculations of secondary structure show that this region is also helical in the zfGLP-1/GCGR and furthermore that this helix is amphiphilic (S1 Fig). Conserved amino acids in this region of zfGLP-1/GCGR lie along the hydrophobic face of the helix. This hydrophobic side of the helix is seen in the crystal structures to interact with the peptide and with the cysteine containing beta turn of the N-terminal domain (NECD) of the receptor, suggesting that the specific amphiphilic helical structure in this region may be important to correctly align the ligand. The hydrophilic side faces the solvent, and could potentially be involved in intramolecular contacts and structural rearrangement within the signaling pathway.

The crystal structures of NECD of hGLP-1R in complex with hGLP-1 or exendin(9–39) show two small antiparallel β-sheets (β1 through β5). The first sheet, involving non-ideal strands β1 (Cys62-Asp67) and β2 (Ala70-Gly75), is conserved in the zfGLP-1/GCGR sequence. The second sheet consists of β3, β4 and β5 spanning residues Gly78-Ser84 (β3), His99-Thr105 (β4), and Leu109-Leu111 (β5). Sequences in the second region of this antiparallel β-sheet, β3-β5, are only partially conserved (Fig 3), but this should not change the structure of zfGLP-1/GCGR, because β-bridges are formed between backbone atoms and therefore are sequence independent. The residues that are conserved in this region are involved in structural stability (blue color in Fig 3, Table 2)

Sequence alignments show much less conservation in the three loops seen in the crystal structures of the NECD of hGLP-1R in complex with exendin(9–39) and hGLP-1 (pink color in Fig 3). Loop 1 that begins at the end of the α-helix is between Pro54 and Phe61, loop 2 spans residues Pro86 and Gly98 and connects β3 and β4 strands, and loop 3, between Gln112 and Leu118, is inserted in the segment Leu109-Asp122. The crystal structure of the NECD of hGLP-1R in complex with exendin(9–39) [35] shows that loop 2 and particularly Pro86 at the beginning of loop 2 is important for the structural integrity of NECD of hGLP-1R. Loop 3 in the crystal structure of NECD of hGLP-1R is defined by amino acids Gln112-Leu118. In the zfGLP-1/GCGR (and rGLP-1R), Gln112 is substituted with histidine (Fig 3), maintaining a similar hydrophilicity of this structural segment, according to the Kyte-Doolittle hydropathy score [63], but all other loop 3 amino acids are absent (Fig 3, Table 2). The shape of this loop allows for this type of deletion because the first and last residues in the loop are close together and a single residue (His112) can close the gap.

As shown in Fig 4 the absence of loop 3 and substitution of Trp120 in hGLP-1R with glycine in zfGLP-1R/GCGR should not have an impact on the interactions with GLP-1 and exendin-4. This observation is consistent with our initial characterization of the zfGLP-1/GCGR which showed that it binds zfGLP-1, hGLP-1 and exendin-4 with similar IC_50_s [41]. It was this observation that led to our conclusion that we characterized a hGLP-1R homolog in zebrafish.

The amino acids in four discontinuous receptor segments that form a hydrophobic binding cavity (Leu32, Thr35, Val36, Trp39 in the α-helix, Tyr69 of turn 1, Tyr88-Leu89-Pro90-Trp91 of loop 2, and Leu123) are sufficiently conserved in the zfGLP-1/GCGR, to maintain this hydrophobic surface core (Fig 3, yellow color). The substitutions of Leu32 to glutamine, Leu123 to threonine, Thr35 to leucine and Val36 to glutamine do not alter the hydrophobic nature because the residues central to the core are 35, 39, 69, 88–91 (Fig 3, yellow color and blue and yellow hatching). These substitutions at positions 35–36 are along the hydrophobic face of the amphiphilic helix (S1 Fig), and as discussed earlier, may allow the correct alignment of exendin(9–39) and hGLP-1 to zfGLP-1/GCGR. Gln32, Leu35, Gln36 and Trp39 in the zfGLP-1/GCGR will allow direct interaction with Phe**22 in exendin(9–39) and Phe*22 in hGLP-1 analogous to the interaction of Leu32, Thr35, Val36 and Trp39 with Phe**22 in exendin (9–39) and Phe *22 in hGLP-1 observed in the crystal structure of NECD of hGLP-1R in complex with exendin(9–39) and hGLP-1, respectively.

The ligand specificity of zfGLP-1/GCGR towards exendin-4 observed in our initial characterization of this receptor [41] and in the present study is consistent with our sequence and structural mapping which showed the conservation in zfGLP-1R/GCGR of sequences and structural features that are important for the formation of the binding pocket of exendin(9–39) (Figs 3 and 5) as seen in the crystal structure of the NECD of hGLP-1R in complex with exendin(9–39) [35].

### Side-chain to backbone interactions

Sequence and structural analysis showed that side-chain to back-bone interactions between different TM helices seen in the 7TM crystal structure of hGCGR may have a similar role in the surface expression of zfGLP-1R/GCGR and in stabilizing conformations important for ligand binding. Among those are TM1-TM7 interaction between Ser152^1.50^ and Ser 390^7.47^ in which Ser152^1.50^ in TM1 in hGCGR forms a hydrogen bond with the backbone of Ser390^7.47^ in TM7. Mutating Ser152^1.50^ (Ser 155^1.50^ in the hGLP-1R sequence) to alanine in TM1 [42] significantly impaired cell surface expression and caused a small reduction of receptor affinity for hGLP-1 and exendin-4, while mutation of Ser390^7.47^ (corresponding to Ser 392^7.47^ in hGLP-1R sequence) to alanine (Fig 6) did not significantly change either cell surface expression of the hGLP-1R or cAMP responses mediated through the hGLP-1R [42]. These findings suggest that correct cell surface expression of hGLP-1R and most likely hGCGR and zfGLP-1/GCGR is maintained as long as Ser152^1.50^ in TM1 can form a hydrogen bond with the backbone of an amino acid at position 390^7.47^ in hGCGR/ 392^7.47^ in hGLP-1R in TM7.

Side-chain to backbone interaction of Asn 318^5.50^ with Leu 242^3.47^ and Leu243^3.48^ seen in the 7TM crystal structure of hGCGR was found to be important for ligand binding of hGLP-1R. Thus, mutation of Asn318^5.50^ to alanine in TM5 in hGLP-1R reduced the receptor’s affinity for hGLP-1, exendin-4 and exendin(9–39), but did not change its cell surface expression [42]. Asn318^5.50^ is found in the corresponding positions in zfGLP-1/GCGR suggesting that it plays a role in the binding affinity of zfGLP-1/GCGR towards GLP-1 and exendin-4 observed in our initial functional characterization of the zfGPCR [41] and in this study.

### Hydrogen bond networks

Our structural and functional analyses suggest that the predicted hydrogen bond network in the NECD of the zfGLP-1/GCGR coordinated by Asp67 (Fig 5), may be engaged in the formation of its binding pocket for hGLP-1, zfGLP-1, exendin-4, exendin(9–39), human glucagon and zebrafish glucagon, analogous to the role of the hydrogen bond network in hGLP-1R [35, 36] and hGCGR [32], respectively. It is likely that in the zfGLP-1/GCGR, the function of this hydrogen bond network is to facilitate the interaction of Lys121 with the main chain atom of peptide residue 27 (Fig 5), i.e. valine in hGLP-1, lysine in zfGLP-1, exendin-4 and exendin(9–39) and methionine in zebrafish glucagon and human glucagon (Fig 1).

The TM3-TM2-TM6-TM7 hydrogen bond network identified in the 7TM structure of hGCGR [30] is coordinated by Glu245^3.50^ in TM3 (Fig 7) and, as shown by mutational analysis of the hGLP-1R, is important for its cell surface expression [42]. The structural similarities with the zfGLP-1/GCGR suggest that this hydrogen bond network may also be critical for the cell surface expression of the dual zfGLP-1R/GCGR.

There likely are similarities with slight differences in the arrangement of the TM4-TM3-TM6 helices between the zfGLP-1/GCGR, hGLP-1R and hGCGR (Fig 8). TM4-TM3 helices in the zfGLP-1/GCGR and hGCGR are potentially closer to TM6 than in hGLP-1R. As a result, TM4-TM3-TM6 helical bundle would be more compact in the zfGLP-1 R/GPCR and hGCGR than in the hGLP-1R (Fig 8). The compactness of the TM4-TM3-TM6 helices may have an impact on the movements of the helical bundle during the conformational changes within the 7TM domains of these receptors upon binding to their respective ligands.

### Structure of extracellular loops

Very little sequence conservation is apparent between the zfGLP-1/GCGR, hGLP-1R and hGCGR in the three extracellular loops ECL1 through ECL3 connecting TM2-TM3, TM4-TM5, TM6-TM7 helices, respectively (Fig 2), except that they are similar in length. Two independent models incorporating results from photoaffinity and mutational experiments, one describing interactions of human glucagon with hGCGR [30] and a second one describing interactions between hGLP-1 and hGLP-1R [56] identified ECL2 and in particular the same Trp295 residue (notation numbered according to the 7TM crystal structure of hGCGR) as a contact amino acid with Leu14, conserved in both human glucagon and in hGLP-1 (Fig 1). These observations suggest that this interaction represents a common structural feature that stabilizes hGLP-1R and hGCGR conformations upon ligand binding. This Trp295 is also conserved in the corresponding position in the zfGLP-1/GCGR, as is Leu14 in the sequences of the zfGLP-1 and zebrafish glucagon (Fig 1); therefore this interaction with ECL2 in the zfGLP-1/GCGR may also stabilize its interaction with the middle regions of the peptides. These and other models [55] also identified other potential contact amino acids in ECL2 as well as in ECL1 and ECL3 [56, 64–66]. Several of these are conserved in the zfGLP-1/GCGR indicating that ECL1, ECL2 and ECL3 may also be important in interactions with amino acids in the N-terminal and middle regions of zfGLP-1, hGLP-1, zebrafish glucagon and human glucagon sequences.

### Loop 3 and the stalk regions

Our sequence and structural mapping highlights a great number of structural features that hGLP-1R shares with hGCGR and that are also found zfGLP-1R/ GPCR. However, in contrast to zfGLP-1/GCGR, with its dual ligand specificity towards both GLP-1 and glucagon, hGLP-1R and hGCGR bind only their respective ligands at physiological concentrations. Sequence alignments between the three receptors show very little sequence conservation in the region representing loop 3, identified in the crystal structures of the NECD of hGLP-1R in complex with either exendin(9–39) [35] or hGLP-1 [36] to be located between Gln112-Trp120 in the hGLP-1R, and Arg112-Trp120 in hGCGR (Fig 3). Loop 3 was also seen in the crystal structure of the NECD of hGCGR [32] and is one amino acid shorter than loop 3 in hGLP-1R (Fig 3). In hGCGR, there is unusual type I turn between Gly109 and Gly112 not seen in the crystal structures of hGLP-1R in complex with either exendin(9–39) [35] or hGLP-1 [36]. In contrast, loop 3 is absent in the sequence of zfGLP-1/GCGR (Figs 3 and 5) yet this does not interfere with the receptor’s ability to bind and be activated by the various peptides.

Another difference between the three receptors is in the C-terminal ends of their NECDs preceding the TM1 helix, also termed the stalk region (Fig 3) that connects the NECDs of these receptors with their 7TM domains. In the 7TM crystal structure of hGCGR, this sequence of 12 amino acids is helical [30]. It has been proposed that the α-helical stalk region in hGCGR may be important in orienting the NECD of the hGCGR towards its transmembrane domain, a rearrangement necessary for ligand induced receptor activation and thus may represent a region which determines the specificity of hGCGR for its ligand, glucagon [30]. In the zfGLP-1/GCGR and in hGLP-1R, the stalk region (which is shorter than in hGCGR) may have the same function, although they may not have the same helical structure as the stalk in hGCGR.

These observations suggest that loop 3 and the stalk region together may contribute to the ligand specificities of the receptors toward their respective ligands. In the crystal structures of the NECD of hGLP-1R in complex with either exendin(9–39) [35] or hGLP-1 [36] loop 3 is flexible and in close proximity to the C-terminal amino acids in hGLP-1. However, the last two C-terminal amino acids in hGLP-1 were not resolved in the crystal structure and therefore their contacts with amino acids in the NECD of hGLP-1R could not be identified and may depend on the presence of the stalk to complete the interaction.

It is likely that binding of hGLP-1 to the full length hGLP-1R may induce a stepwise change in conformational states to present the peptide to the TM of the receptor via a more structured conformation of loop 3, which may take place in the full length hGLP-1R after the initial alignment of the C-terminal end of hGLP-1 to the amphiphilic helix in the NECD (S1 Fig) and upon contact between the main chain atom of peptide residue 27 (Val*27 in hGLP-1) and Arg121 in hGLP-1R coordinated by Asp67 in the hydrogen bond network (Fig 5). Loop 3 may then facilitate the alignment of the N-terminal end of hGLP-1 in a specific orientation towards the stalk which may then present hGLP-1 to the 7TM domain, allowing hGLP-1 to make new contacts through the amino acids in the middle region of its sequence with the full length hGLP-1R and in particular the ECL loops stabilizing a new hGLP-1R conformation. Finally, insertion of His*1 into the inter-helical regions may trigger additional conformational change(s) through the movements of the helical bundle leading to hGLP-1R activation across the membrane. This is likely a critical step as found in the early structure-function studies where deletion of His*1 abolished the binding to rGLP-1R [12]

Contribution of the C-terminal amino acids in GLP-1 to its binding to the rGLP-1R and receptor activation was observed in our early structure-function experiments showing that a sequential deletion of the C-terminal Arg*30, Gly*29 and Lys*28 (Fig 1) led to a gradual loss of binding [12], cAMP responses and stimulation of insulin secretion [67]. A truncated GLP-1 analog, in the absence of the last five C-terminal residues (Val*27-Lys*28-Gly*29-Arg*30-Gly*31), was unable to stimulate insulin secretion from the perfused rat pancreas [67]. These early observations are consistent with the crystal structure of the NECD of hGLP-1R in complex with hGLP-1 [36]. They also highlight that the interaction of Val*27 in GLP-1 with Arg121 in the hydrogen bond network coordinated by Asp67 in hGLP-1R (Fig 5), together with the four C-terminal Lys*28-Gly*29-Arg*30-Gly*31 residues, are critical for stabilizing a GLP-1R conformation that leads to an active state.

Loop 3 and the stalk region in hGCGR may have a similar function in conferring its ligand specificity towards glucagon by facilitating the contacts between amino acids in its N-terminal and middle region of glucagon with the 7TM domain of hGCGR. Deletion of the last three C-terminal residues in glucagon abolished its biological activity [68] suggesting that, as in GLP-1, the last three C-terminal amino acids in glucagon may be critical for stabilizing a hGCGR conformation that leads to an active state.

The dual specificity of the zfGLP-1/GCGR towards GLP-1 and glucagon may be a consequence of the absence of loop 3 (Figs 3 and 4). Without loop 3, the fine-tuning of the specific orientations of GLP-1 and glucagon towards the stalk region of the zfGLP-1/GCGR may be lost. Instead, amino acids in the N-terminal and middle regions of zfGLP-1, hGLP-1, zebrafish glucagon and human glucagon may form similar contacts with the amino acids in ECL1, ECL2 and ECL3 loops and TM helices in the zfGLP-1/GCGR and stabilize similar conformation(s) leading to its active state.

In summary, the dual zebrafish GLP-1R/GCGR contains many of the structural elements found in the hGLP-1R and hGCGR and its further characterization will contribute to our understanding of the ligand-induced conformations within hGLP-1R and hGCGR structures that influence the specific recognition and activities of their ligands. It also poses a question about the type of selection pressures that guided the emergence of highly specific receptors for glucagon and GLP-1 in the lineage leading to mammals and the emergence of a receptor with dual ligand selectivity towards GLP-1 and glucagon in the lineage leading to zebrafish.

## Supporting Information

S1 Fig A helical wheel representation [46] highlighting the amphiphilic nature of the helix formed by residues 31–52 in the sequence of hGLP-1R, and the analogues sequences in zfGPCR (dual zfGLP-1R/GCGR) and hGCGR. The hydrophobic patch (greens/yellows) on the right side of the helix is maintained in zfGPCR (zfGLP-1R/GCGR) and hGCGR while the hydrophilic patch (blues/reds) is slightly perturbed.
(TIF)
