# Supplementary figures and images for: Structural Mapping and Functional Characterization of Zebrafish Class B G-Protein Coupled Receptor (GPCR) with Dual Ligand Selectivity towards GLP-1 and Glucagon
Source: PLoS One. 2016 Dec 8;11(12):e0167718. doi: 10.1371/journal.pone.0167718 (PMC5145181; doi:10.1371/journal.pone.0167718)

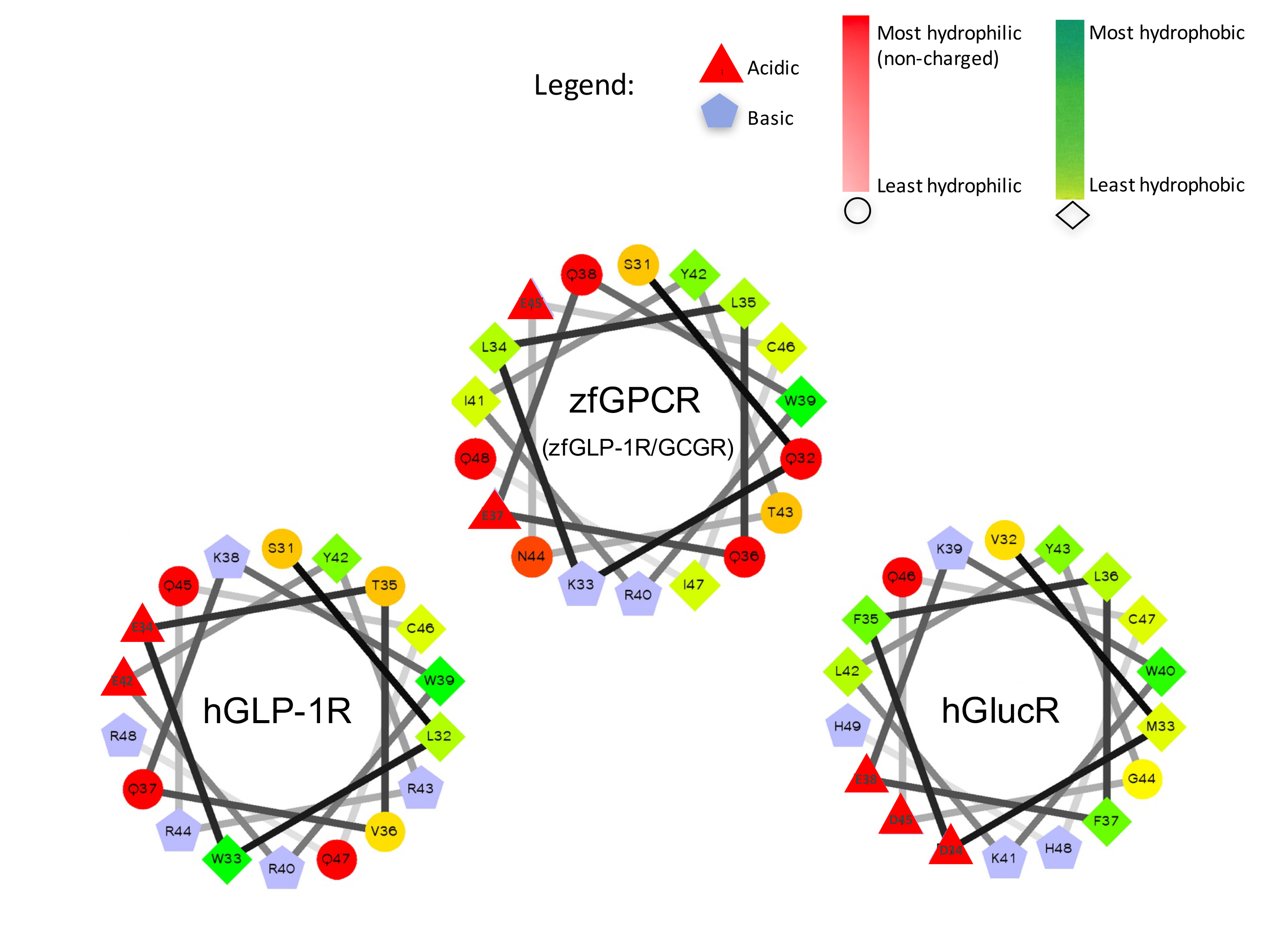

Supplement: S1 Fig — The hydrophobic patch (greens/yellows) on the right side of the helix is maintained in zfGPCR (zfGLP-1R/GCGR) and hGCGR while the hydrophilic patch (blues/reds) is slightly perturbed. (TIF) [file pone.0167718.s001.tif]
